# Correlated Motions of Conserved Polar Motifs Lay out a Plausible Mechanism of G Protein-Coupled Receptor Activation

**DOI:** 10.3390/biom11050670

**Published:** 2021-04-30

**Authors:** Argha Mitra, Arijit Sarkar, Márton Richárd Szabó, Attila Borics

**Affiliations:** 1 Laboratory of Chemical Biology, Institute of Biochemistry, Biological Research Centre, Szeged, 62. Temesvári krt., H-6726 Szeged, Hungary; argha.mitra@brc.hu (A.M.); sarkar.arajit@brc.hu (A.S.); szabo.marton@med.u-szeged.hu (M.R.S.); 2 Department of Biochemistry, Faculty of Medicine, University of Szeged, 9 Dóm sq., H-6720 Szeged, Hungary

**Keywords:** GPCR, opioid, activation mechanism, signal transduction, molecular dynamics

## Abstract

Recent advancements in the field of experimental structural biology have provided high-resolution structures of active and inactive state G protein-coupled receptors (GPCRs), a highly important pharmaceutical target family, but the process of transition between these states is poorly understood. According to the current theory, GPCRs exist in structurally distinct, dynamically interconverting functional states of which populations are shifted upon binding of ligands and intracellular signaling proteins. However, explanation of the activation mechanism, on an entirely structural basis, gets complicated when multiple activation pathways and active receptor states are considered. Our unbiased, atomistic molecular dynamics simulations of the μ opioid receptor (MOP) revealed that transmission of external stimulus to the intracellular surface of the receptor is accompanied by subtle, concerted movements of highly conserved polar amino acid side chains along the 7th transmembrane helix. This may entail the rearrangement of polar species and the shift of macroscopic polarization in the transmembrane domain, triggered by agonist binding. Based on our observations and numerous independent indications, we suggest amending the widely accepted theory that the initiation event of GPCR activation is the shift of macroscopic polarization between the ortho- and allosteric binding pockets and the intracellular G protein-binding interface.

## 1. Introduction

G protein-coupled receptors (GPCRs) are located on cell surfaces and act as communication interfaces for external stimuli exerted by structurally diverse molecules. Upon activation, GPCRs initiate signal transduction through interactions with G proteins and arrestins, and control a variety of intracellular processes. Owing to this, approximately 34% of all prescription pharmaceuticals target members of this receptor family [1]. However, application of such drugs is often limited by a number of unwanted side effects due to non-selective activation of multiple GPCRs, or multiple signaling pathways associated with one receptor. The most recent challenge of rational drug design is, therefore, to develop signaling pathway-specific, or in other words “functionally selective” GPCR agonists. To address this challenge, complete understanding of the structural mechanism of GPCR activation is necessary. Opposed to the high diversity of external activators, signaling is mediated by only a few types of G proteins, advocating that GPCR activation may follow a general mechanism. 

The structure of GPCRs consists of a conserved bundle of seven transmembrane (TM) α-helices, and highly dynamic extracellular and cytosolic domains of various lengths. High-resolution experimental structures are available for many GPCRs both in the active and inactive states (for a comprehensive collection visit http://gpcrdb.org, accessed on 28 April 2021) [2], but the mechanism of transition between these forms is intensely debated. The most conspicuous difference between the active and inactive class A GPCR structures, published to this date, is a notable disposition of the 6th transmembrane helix (TM6) [3,4]. However, such large dispositions were shown to occur even in the absence of a bound ligand, due to the inherent dynamics of the receptor structure, or may originate from the applied conditions of crystallographic structure determination, namely the attachment of fusion proteins or the application of crystallization chaperones [5,6,7]. Apart from TM6 disposition, a possible role of intracellular loop 1 (ICL1) and the cytosolic helix (H8) in the activation mechanism was highlighted by dynamic NMR measurements of the μ-opioid receptor (MOP) [8]. Comparison of the structures of active and inactive state MOP and δ-opioid (DOP) receptors suggested that an extended network of polar amino acids and water molecules connects the orthosteric ligand binding pocket to the cytosolic domains, which may be functionally relevant. The highly conserved polar functional motifs, E/DRY, NPxxY, and CWxP, have been specified to participate in the activation mechanism of class A GPCRs and the known effect of elevated concentrations of Na^+^ that prevents the agonist-induced activation of opioid receptors [9] and related GPCRs was attributed to a conserved allosteric Na^+^ binding site [10,11,12,13,14]. Conceivably, activation signal is transmitted to the intracellular surface of the receptor through the interplay of these polar microswitches, however, no direct evidence of such integral mechanism has yet been given. Real-time observation of such processes using conventional experimental techniques is unattainable. 

A significant part of the now widely accepted theory of GPCR activation was provided by landmark molecular dynamics (MD) simulation studies [3,4,13,14]. According to this theory, GPCRs exist as a dynamic ensemble of multiple active, inactive, and intermediate states. The populations of active states are increased by agonist binding and the stabilization of an active structure facilitates the insertion of G proteins [13]. The growing amount of evidence of pre-coupled GPCR-G protein complexes in the absence of ligands, however, presents a challenge to the above hypothesis [15]. In general, explanations given on an entirely structural basis are often diffuse and fail to provide unequivocal suggestion for a possible structural mechanism of GPCR activation, especially when multiple active states, or structurally similar but functionally different ligands are considered. Further limitations of previous MD studies are that simulation systems were confined to the TM region of GPCRs, embedded in very simplistic representations of the cell membrane. Most recently, special effects of charged interfacial lipids on β_2_-adrenergic receptor signaling was demonstrated, drawing attention to the importance of accurate membrane representation [16]. 

The MOP is one of the most extensively studied GPCRs, therefore it appropriately represents the general structural features of class A (rhodopsin-like) GPCRs. In order to get a deeper insight, we have performed all-atom MD simulations of the full sequence MOP, including the N- and C-terminal domains, on a μs timescale. To better approximate the physiological conditions of the activation mechanism, simulations of the active and inactive receptors were executed in caveolar membrane environment [17], in the presence of the endogenous agonist endomorphin-2 (EM2, H-Tyr-Pro-Phe-Phe-NH_2_) [18] and the G_i_ protein complex, or beta-arrestin-2. In addition, reference simulations were carried out in the presence of allosterically bound Na^+^ or in the absence of EM2. Further control simulations were performed with fused T4-lysozyme or intracellularly bound Nb39 nanobody, representing the previously applied crystallization conditions [5,7]. Our simulations were intended to gather information about how the N- and C-terminal domains, intracellular proteins, and crystallization chaperones affect the internal dynamics of the TM domain and consequently the activation mechanism. The analysis of trajectories was aimed at confirming the integrity of the simulation systems, comparing structural properties of our systems to previously published simulation data, as well as introducing new perspectives.

## 2. Methods

### 2.1. System Building

The crystallographic structures used in this study were downloaded from the Brookhaven Protein Data Bank (http://www.rcsb.org, accessed on 28 April 2021): active MOP (pdb code: 5C1M), inactive MOP (pdb code: 4DKL), heterotrimeric G_i_ protein complex (pdb code: 1GP2), Nb39 nanobody (pdb code: 5C1M), T4-lysozyme (pdb code: 4DKL), β_2_-adrenergic receptor complexed with G_s_ protein (pdb code: 3SN6), and rhodopsin complexed with beta-arrestin-2 (pdb code: 4ZWJ). These latter two structures were used as templates to orient the G_i_ protein complex and beta-arrestin-2 to the active and inactive MOP. The full sequence of the murine MOP (UniProtKB-P42866-OPRM1) was obtained from UniProt (http://www.uniprot.org, accessed on 28 April 2021), and the coordinates of the membrane orientation from the OPM server (http://opm.phar.umich.edu, accessed on 28 April 2021). The crystallization chaperone and fusion protein (Nb39 nanobody and T4-lysozyme, respectively) were removed from the crystallographic structures. The Swiss-PdbViewer was used to retrieve all missing, modified, or mutated residues of the transmembrane (TM) domain of the receptor. GTP was generated in CHARMM-GUI [19] and edited manually to replace GDP in the G_i_ complex. 

To model the missing N- and C-terminal domains, 10 ns folding simulations of the N- and C-terminal domains were performed using the GROMACS ver. 5.1.4 program package [20], the AMBER ff99SB-ILDN-NMR [21] force field, and the GB/SA implicit solvation model [22]. During MD simulations, the system temperature was set to 310 K and maintained by the v-rescale algorithm [23]. Ten parallel simulations were run for both the N-and C-terminal domains from where the resultant, folded structures were evaluated and selected based on the compactness, accessibility of post-translational modification, and TM region attachment sites. (For further details, see the “MD trajectory analysis” subsection below.) Glycosylation sites were predicted using the NetNGlyc 1.0 online server [24]. The selected N-and C-terminal domain structures were linked to the TM region using Pymol ver. 2.1.0. Four intracellular partners were used in this study, namely the heterotrimeric G_i_ protein, beta-arrestin-2, Nb39 nanobody, and T4-lysozyme. Among them the last two were used for reference simulation systems. The first three proteins were attached non-covalently to the receptor, while T4-lysozyme was fused with the receptor replacing the third intracellular loop (ICL3), similar to that in the crystallographic structure of the inactive MOP (pdb code: 4DKL). Cryo-electron microscopic structure of the G_i_ protein-bound MOP [6], published later, have verified the adequacy of the corresponding model built in this study (Figure S1). 

CHARMM-GUI was used to include various post-translational modifications, as well as to build membrane bilayers. Complex type glycans were added to the N-terminal domain, containing a common core (Manα1-3 (Manα1-6) Manβ1- 4GlcNAcβ1-4GlcNAcβ1-N) and sialic acid (N-acetylneuraminic acid) at glycosylation prone N9, N31, and N38 residues of the N-terminal domain [25]. Phosphorylation of S363 and T370 were done for all the complexes, while S375, T376, and T379 sites at the C-terminal domain were phosphorylated in addition for the arrestin complexes [26]. The C170 residue of ICL2 was palmitoylated [27].

A caveolar membrane environment, considered to be the physiological environment of the MOP, was built using the membrane builder tool of CHARMM-GUI. CHARMM36 parameters were used to build complex, multicomponent membrane systems, which included cholesterol (CHL-32.8%), 1-palmitoyl-2-oleoyl-glycero-3-phospho-choline (POPC-14.9%), 1-palmitoyl-2-oleoyl-sn-glycero-3-phosphoethanolamine (POPE-27.8%), 1-palmitoyl-2-oleoyl-sn-glycero-3-phospho-L-serine (POPS-3.6%), 1-palmitoyl-2-oleoyl-sn-glycero-3-phosphoinositol (POPI2-6%), palmitoyl-sphingomyelin (PSM-9.9%), and monosialodihexosylganglioside (GM3-5%) [17]. GM3 gangliosides were generated separately using the glycoprotein builder tool of CHARMM-GUI, and then added manually to the membrane. The asymmetric upper and the lower leaflet membrane compositions were specified in a most probable ratio [28]. The membrane builder was also used to embed the glycosylated, palmitoylated, and phosphorylated full sequence model of the MOP into the membrane. Systems were then solvated explicitly with TIP3P water molecules in a hexagonal shaped periodic box, and sodium and chloride ions (0.15 M) were added to neutralize the net charge and to attain physiological ionic strength. System coordinates and topologies were generated in GROMACS format.

EM2 [18], a peptide agonist of the MOP was built manually, using Pymol ver. 2.1.0. The binding site was confirmed by flexible docking of this ligand to the active state MOP crystallographic structure (pdb code: 5C1M), using the Autodock ver. 4.2 software [29] and the Lamarckian genetic algorithm. All φ, ψ, and χ^1^ ligand torsions, as well as receptor side chains in contact with the bound ligand [5] were kept flexible. Docking of EM2 was performed in an 8.0 nm × 8.0 nm × 8.0 nm grid volume, large enough to cover the whole binding pocket of the receptor region accessible from the extracellular side. The spacing of grid points was set at 0.0375 nm and 1000 dockings were done. The resultant ligand-receptor complexes were clustered and ranked according to the corresponding binding free energies. The lowest energy bound state was selected for simulations, in which specific ligand-receptor interactions observed in the crystallographic structures were present. Cryo-electron microscopic structure of the MOP and the peptide agonist DAMGO, published later, have confirmed the correct localization and analogous orientation of pharmacophores of EM2 [6]. Two additional control simulation systems were built for the active state, G_i_ protein-bound receptor, either with the exclusion of bound EM2, or with the inclusion of EM2 in the orthosteric site together with a Na^+^ ion placed in the proximity of D114^2.50^ (Ballesteros-Weinstein numbering is indicated in the upper index) in the allosteric binding pocket [10,11,12,13,14]. 

### 2.2. MD Simulations

All equilibration and production MD simulations were performed using the GROMACS ver. 5.1.4. molecular dynamics program package. Ten independent simulations were performed, four each for inactive and active MOP, complexed with heterotrimeric G_i_ protein, beta-arrestin-2, Nb39 nanobody, and T4-lysozyme. Additionally, reference simulations were run for the ligand-free, active state, G_i_ protein-bound receptor, and for the EM2-bound active receptor-G_i_ protein complex in the presence of an allosteric Na^+^ ion. After orienting and adding EM2, where applicable, the resultant complex systems were energy minimized thoroughly performing 5000 steps steepest descent, followed by 5000 steps conjugate gradient minimization with convergence criteria of 1000 kJ/mol nm^-1^ in both cases. After minimization, systems were subjected to a six-step equilibration protocol, supplied by CHARMM-GUI. According to this protocol, positionally restrained MD simulations were executed in the canonical (NVT) and then, after 2 steps, in the isobaric-isothermal (NPT) ensemble at 303.15 K and 1 bar, having the positional restraints on the heavy atoms of the proteins and membrane constituents decreasing gradually. The first three equilibration MD runs were done for 25 ps in 1 fs time steps, and the following two were continued for 100 ps in 2 fs time steps. The sixth step of the equilibration protocol was run for 50 ns in 2 fs time steps. The following, further parameters were applied: the LINCS algorithm was used to constrain all bonds to their correct length, temperature was regulated by the v-rescale [23] algorithm with a coupling constant of 1 ps, semi-isotropic Berendsen pressure coupling [30] was applied with a coupling constant of 5 ps and compressibility of 4.5 × 10^−5^ bar^−1^. The Particle Mesh Ewald (PME) method was used and a twin-range cutoff was applied to calculate energy contributions from electrostatic and van der Waals interactions, respectively. All cut-off values were set to 1.2 nm. After equilibration, production simulations were performed for 1 μs at 310 K in the NPT ensemble, with other parameters same as above. The coordinates were stored in every 5000th steps yielding trajectories of 100.000 snapshots.

### 2.3. MD Trajectory Analysis

Analysis of MD trajectories was performed using the analysis suite of the GROMACS 5.1.4 package. Generally, the analyses of MD trajectories were performed to evaluate membrane properties and protein conformational changes, stability of the molecular complexes, as well as to investigate previously described interactions and their role in different activation states of the receptor. 

Folding simulations were assessed through the analysis of root mean square deviation (RMSD) of backbone atom positions with respect to the starting structures. Furthermore, the radius of gyration of the N- and C-terminal domains were measured, and the number of intramolecular H-bonds were calculated along the trajectories using the gmx gyrate and gmx hbond utilities, respectively. The evolution of secondary structure was monitored using the DSSP (Define Secondary Structure of Proteins) method [31]. Membrane thickness and area per lipid head-group values were calculated using the FATSLiM 0.2.1 program [32].

For each production MD trajectory RMSD, calculations were carried out to assess the structural stability of the complex and demonstrate significant displacements of structural components as a function of time. RMSD values of protein backbone atoms were calculated in comparison with the active and inactive state starting structures. The dynamics of terminal domains during simulations was examined by monitoring the radii of gyration. Conformational fluctuations of specific amino acid side chains were analyzed by measuring side chain χ^1^ angles and calculating the frequency of transitions between rotameric states using gmx chi. The gmx helix utility was used to calculate helix properties. Secondary structure assignment was, again, done using the DSSP method.

The occurrence and frequency of intra- and intermolecular H-bonds were calculated using the gmx hbond utility. The donor-acceptor distance and donor-hydrogen-acceptor angle cut-offs for H-bond assignments were set to 0.35 nm and 30.0 degrees, respectively. The presence of salt bridges was monitored by measuring distance and angle between the corresponding acidic and basic side chain functional groups, using gmx distance and gmx angle, respectively. The distance threshold for salt bridge assignment was 0.4 nm and the angle threshold was 90.0 degrees. Where applicable, penetration of Na^+^ ions into the allosteric Na^+^ binding site, D114^2.50^, was checked using the gmx mindist utility.

The extent of correlation of atomic displacements was examined by dynamic cross- correlation matrix analysis (DCCM) integrated into an earlier version of the GROMACS suite (g_correlation, ver. 3.3) [33]. The GIMP ver. 2.8 software was used for image analysis of the obtained DCCM maps, where the extent of correlation was demonstrated by color intensity. The threshold of assignment of correlation was red color intensity corresponding to >0.7 MI (mutual information). Amino acid side chains having at least 4 atoms participating in correlated motions were considered. The threshold of 4 atoms have been set in order to exclude irrelevant sidechain motions, such as torsional rotations of methyl groups.

Systems were visualized using Pymol ver. 2.1.0 or VMD ver. 1.9.3. software [34] and graphs were prepared using the Xmgrace ver. 5.1.25 program.

### 2.4. Sequence Alignment and Conservation Analysis

244 sequences of class A mouse GPCRs (without orphan and olfactory receptors) were retrieved from the UniProt database in FASTA format. The Clustal Omega program [35] was used to align those multiple sequences and the results were analyzed using Jalview ver. 2.10.5. [36] The OPRM_MOUSE (P42866) sequence was set as reference. The sequences were compared based on percentage of identity.

## 3. Results and Discussion

### 3.1. System Building

An initial challenge in this study was to build simulation systems which approximate the physiological conditions of the MOP as closely as possible. The N- and C-terminal domains of the receptor were included to account for the drag posed by the mass of these domains and its effect on the dynamics of transmembrane helices, of which the central role in the activation mechanism was widely emphasized by previous proposals [3,4,5,6,7,8,12,13,14]. In the absence of atomic resolution structures of these or homologous domains, folding simulations were performed to create approximate structures. Although the evolution of backbone RMSD, radius of gyration, number of H-bonds, and secondary structure indicated convergence of folding simulations in most cases (Figure S2), it is ambiguous that the folding of N- and C-terminal domains were correct and complete in the given time frame. Nevertheless, the aforementioned primary purpose of the inclusion of these domains was sufficiently fulfilled by the generated structures. Partial unfolding of the N- and C-terminal domains was observed during some of the production simulations (Figure S3), but the size of the periodic box, varying between 17.64 nm and 24.00 nm in the Z dimension was large enough to keep periodic images of these domains from contacting each other and to produce artifacts (Figure S4).

### 3.2. Membrane Properties

Immediate vertical contraction of the membrane was observed in the equilibration phase, approaching the thickness of homogeneous phospholipid bilayers [37], whereas lateral contraction was more gradual (Figure S5). Then, after the removal of all restraints, a slight vertical expansion of the membrane was observed in the initial production phase (first 20–30 ns), then the membrane stabilized at approximately 4.30 nm thickness, a value more appropriate for lipid rafts [38]. Lateral contraction of the membrane continued in the initial production phase until stabilization at approximately 0.49 nm^2^ after 30 ns. It is important to note, that the initial imbalance of membrane parameters in the production phase emerge from that positional restraints on the protein were released only at the beginning of the production phase. Since simulations were intended to monitor the process of activation, of which timescale could not be estimated, the possibility of structural changes in the equilibration phase had to be kept minimal. Therefore, positional restraints on the heavy atoms of the protein were decreased step-by-step, but maintained throughout the equilibration. No artificial trends reflecting this initial membrane imbalance were perceived in the results. The average values for bilayer thickness and area per lipid headgroup, having excluded the values of the first 40 ns, were 4.328 nm and 0.493 nm^2^, respectively.

### 3.3. Allosteric Na^+^ Binding

Penetration of Na^+^ ions into the allosteric Na^+^ binding site (D114^2.50^) did not happen during the simulations of EM2-bound receptors (Figure S6), regardless of the receptor state. While H_2_O molecules were observed to exchange between the internal cavities of the transmembrane domain as well as the bulk solvent phase, Na^+^ ions did not enter the TM domain, neither from the extracellular nor the intracellular side. This suggests that for Na^+^ ions the allosteric site is only accessible through the orthosteric binding pocket, and its entrance could be blocked by a bound ligand, whereas intracellular access to the TM domain is closed by the G_i_ protein. Conversely, Na^+^ quickly localized at the allosteric site when EM2 was not present, following the transition of the receptor to an intermediate structural state. Interestingly, Na^+^ binding to the orthosteric anchor residue, D147^3.32^, was significantly less frequent, rather occasional in the ligand-free receptor. Moreover, binding of Na^+^ to the orthosteric site and dissociation of Na^+^ from the allosteric site was often coincidental (Figure S7a). The very low frequency of simultaneous occupation of ortho- and allosteric sites, however, may be simply the consequence of the applied Na^+^ concentration and more frequent simultaneous occupation could be achieved at higher ionic strengths. The observed spontaneous Na^+^ penetration form the extracellular environment is in agreement with previously published MD simulation data [39,40], but this and the occupancy data presented above is insufficient to provide explanation for the modulation of receptor activation. The localization of Na^+^ ions in the transmembrane region and their effect on the activation of class A GPCRs have been extensively studied previously, both by experimental and theoretical methods [10,11,12,39,40,41]. Translocation of Na^+^ ions through the active state MOP was observed in previous MD simulations, but in the absence of bound ligands and intracellular proteins [41]. According to the current state-of-the-art receptor activation is accompanied by the “collapse” of the allosteric Na^+^ binding site, which results in the ejection of the bound Na^+^ and its migration towards the cytosol. [41]. Our simulation of the active MOP-G protein complex in the presence of EM2 at the orthosteric binding site, and Na^+^ placed initially at the allosteric Na^+^ binding site, have provided corroborating results. Compared to the ligand-free system, the localization of the Na^+^ ion in the allosteric site and its contacts with D114^2.50^, S154^3.39^, and W293^6.48^ have become loose during the simulation. Consequently, the frequency of close contacts with conserved polar/amphipathic residues further down towards the intracellular surface (N328^7.45^, N332^7.49^, and Y336^7.53^) have increased significantly (Figure S7b).

### 3.4. TM Helix and Loop Dynamics

Atomic displacement analysis of transmembrane helical backbones of the EM2-bound receptors indicated, that TM6 assumed intermediate conformations during simulations with minor changes from the corresponding starting structures (Figure S8). This is in line with previous simulation results, where notable TM helix rearrangements were only observed at longer timescales and in the absence of bound intracellular proteins [13]. 

**Figure 1:**
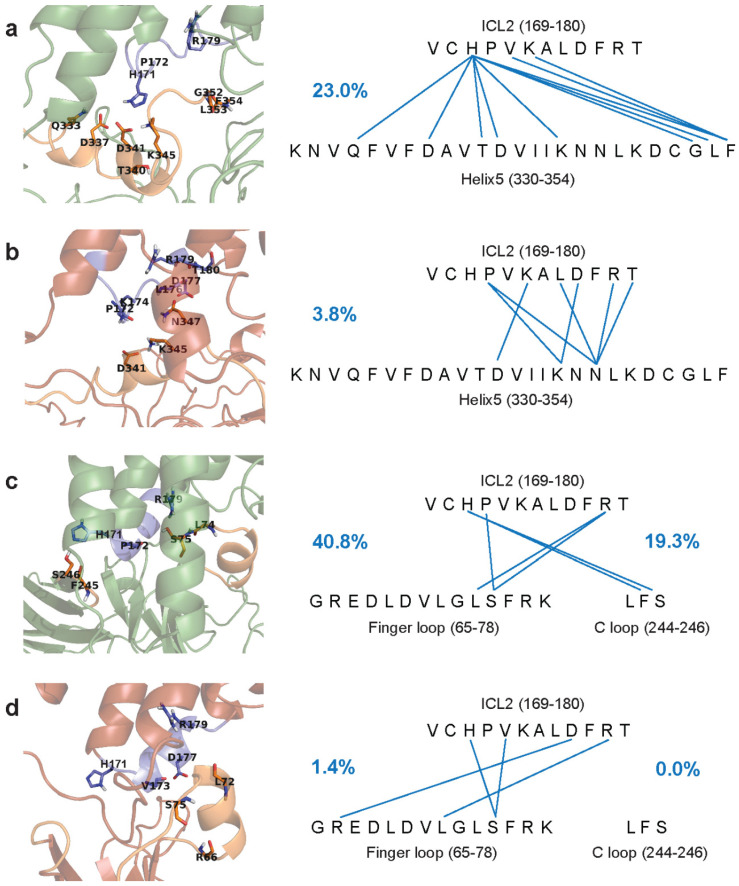
Frequency and donor and acceptor sites of intermolecular H-bonds between ICL2 of the MOP and the G_i_ protein or beta-arrestin-2. Frequency of H-bonds are expressed as percentages of the total structural ensemble and indicated by blue numbers. ICL2 is shown as blue cartoon and sticks. Helix 5 of the G_i__α_ subunit and the finger- and C loops of beta-arrestin-2 are shown as orange cartoon and sticks. (**a**) Active receptor and the G_i__α_ subunit. (**b**) Inactive receptor and the G_i__α_ subunit. (**c**) Active receptor and beta-arrestin-2. (**d**) Inactive receptor and beta-arrestin-2.

The largest disposition was measured for the inactive receptor beta-arrestin-2 complex, suggesting a preference of beta-arrestin-2 for the active structural state of the receptor. TM6 of the ligand-free receptor, on the other hand, underwent much larger changes. This demonstrates remarkable stabilizing effect of the agonist, regardless of activation state. Initial conformations of ICL1 and H8 were maintained throughout simulations in each receptor state, regardless of the intracellular interacting partners (Figures S9 and S10). The second intracellular loop (ICL2), on the other hand, adopted a stable α-helical structure when bound by beta-arrestin-2 and partially unfolded upon interaction with the G_i_ subunit, independent of the state of the receptor (Figure S11). This latter, however, was only observed in the presence of EM2 in the orthosteric site and the absence of Na^+^ in the allosteric site. Apparently, this structural transition of ICL2 was prevented by the bound Na^+^ ion. In the active states, increased frequency of intermolecular hydrogen bonds was observed involving ICL2, helix 5 of G_iα_, and the finger- and C-loops of beta-arrestin-2 (Figure 1, Table S1). These observations indicate that the conformations of ICL1 and H8 is controlled by the receptor state whereas, in the presence of an agonist, ICL2 adapts its structure to the bound signaling proteins. Therefore, ICL2 may be partially responsible for signaling pathway specificity. Such dynamics of ICL2 was not indicated by the published high resolution structures of this receptor [5,6,7]. Supporting evidence was, however, provided by a most recent NMR spectroscopic study of the β_2_-adrenergic receptor. Distinct conformations of ICL2 were indicated when the receptor was bound either by the G_s_ or the G_i1_ protein complex and, similar to the results presented here, G_i1_ did not promote the formation of an alpha helix in ICL2 [42]. Peculiar results were obtained for the active, ligand-free, G_i_ protein-bound receptor. ICL2 have folded into an α-helix during simulation, but maintained strong contact with the G_iα_ subunit (Figures S11 and S12). Although H-bonds between the G_iα_ subunit and the receptor involved only one participant residue of ICL2, the frequency of this H-bond indicated stronger and more specific interaction. This observation supports the hypothesis of pre-coupled GPCR-G protein complexes in the absence of ligands [15], and suggests that the lower frequency and specificity of intermolecular H-bonds in the EM2-bound active receptor may represent an intermediate complex state, which precedes G_i_ protein dissociation during the signaling event. Secondary structure analysis of the control systems with Nb39 or T4-lysozyme fusion suggests that these systems better represent the arrestin-bound state of the receptor (Figure S11).

### 3.5. Specific Intramolecular Interactions

Analysis of intramolecular salt bridges and H-bonds between conserved motifs (Table 1) indicated, in agreement with previous proposals [7], that interactions between D164^3.49^ and R165^3.50^ of the DRY motif were more frequent in the inactive states and in the ligand-free receptor. The specific role of the previously reported DRY-TM5 [7], DRY-TM6 [7], and NPxxY-TM [5] contacts in the activation mechanism could not be deduced from our simulation results. No systematic connection was found between the frequency of those interactions and physiologically relevant receptor states and complexes within the time frame of simulations. The frequency of CWxP-TM7 interactions was, however, significantly higher in the ligand-free receptor (Table 1). Furthermore, a salt bridge between R165^3.50^ (DRY) and D340^8.47^ (H8) was found to be present only in the active state and most frequent in the presence of the G_i_ protein complex. This latter specific interaction was not described previously as, opposed to the aforementioned contacts, it was not evidently present in the reported high-resolution structures [5,6,7]. Our data suggest that this contact could be important for receptor activation and it is further supported by earlier mutation experiments [43]. 

### 3.6. Correlated Side-Chain Motions and the Polar Signaling Channel

**Figure 2:**
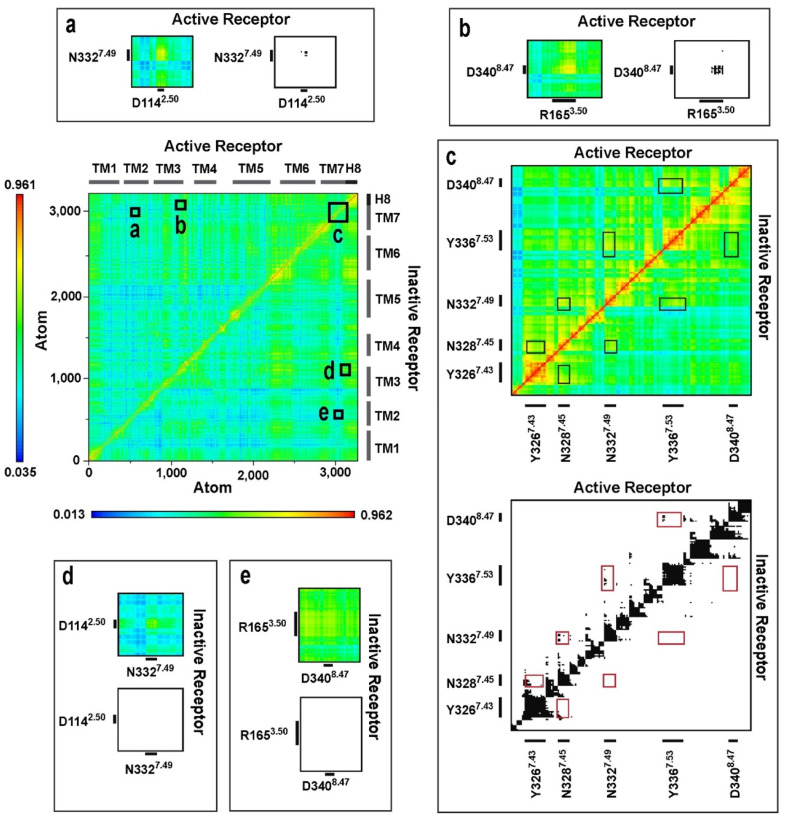
Dynamic cross-correlation matrices of the G_i_ protein-bound MOP in active and inactive states. Panels (**a**–**e**) are magnified views of regions of amino acid residues of interest. Black and white panels show correlations above the threshold of 0.7 MI.

**Figure 3:**
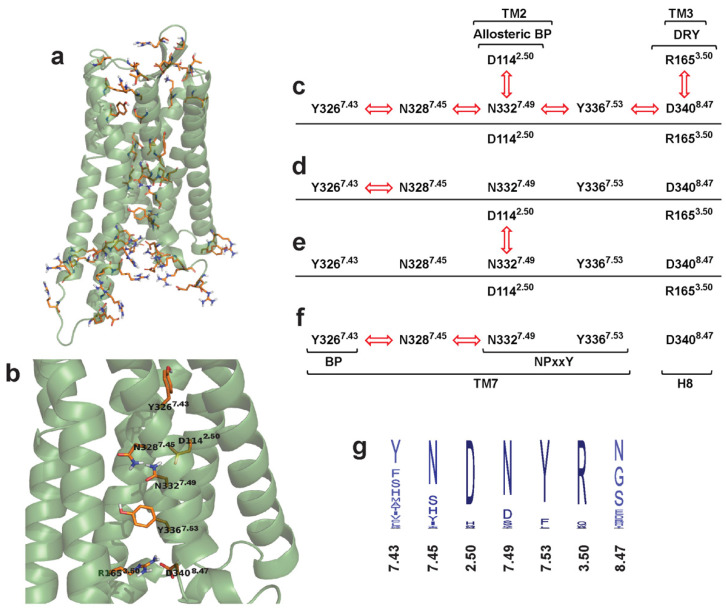
The polar signaling channel of the MOP revealed by dynamic cross-correlation analysis. (**a**) Polar amino acids of which motions are correlated in the G_i_ protein-bound active state. (**b**) Polar amino acids of which motions are correlated and connecting the orthosteric binding pocket to the G protein-binding interface. Diagrams of channel residues in (**c**) the active receptor-G_i_ protein, (**d**) inactive receptor-G_i_ protein, (**e**) active receptor-beta-arrestin-2 and (**f**) inactive receptor-beta-arrestin-2 complexes. Red arrows indicate correlated motions of the respective amino acids. (**g**) Degree of conservation of polar signaling channel residues of class A GPCRs. Non-polar hydrogens are omitted for clarity.

Dynamic cross-correlation analysis of the transmembrane domain and the extra- and intracellular loops provided the most conspicuous results (Figure 2). According to those, the orthosteric binding pocket is connected to the intracellular surface through a channel of polar amino acid residues, of which motions are highly correlated (Figure 3). Such concerted motions were observed only for the active receptor-G_i_ protein complex (Figure 3 and Figures S13–S18), suggesting that this phenomenon has a fundamental role in G protein-mediated signaling. Interestingly, no such concerted motions of these polar amino acid side chains were found in the ligand-free receptor either, although it was expected on the basis of higher conformational flexibility and consequential constitutive activity. The freezing or decoupling of these correlated motions may be attributed to the shift of a positive charge from the orthosteric binding site to the allosteric Na^+^ binding site, or to the notable disposition of transmembrane helices observed during the simulation of the ligand-free receptor. The former potential reason is further supported by the apparent lack of correlated motions in the active MOP-G_i_ protein-EM2-allosteric Na^+^ system, where the Na^+^ ion was shown to connect frequently with the aforementioned channel of polar residues (Figure S7b). On the other hand, both reasons could be debated, considering that neither such charge transfer nor similar TM helix disposition was observed in the other reference systems. Residues of the above identified polar signaling channel are located mostly on the 7^th^ transmembrane helix (TM7), in the inner region of the transmembrane helical bundle, most distant from the surrounding membrane environment. All channel residues are parts of highly conserved functional motifs and allosteric Na^+^ binding sites, except for Y326^7.43^ of the binding pocket and N340^8.47^ at the G protein-binding interface. The increased variability of these two residues is associated with ligand and G protein specificity, respectively. Special attention should be paid to residue Y326^7.43^ of the binding pocket. Similar to highly conserved residues of the binding pocket, such as D147^3.32^, often referred to as the “anchor residue”, this variable residue is always found to be in close contact with the bound ligands in various class A GPCRs. Furthermore, there is a strong complementary relationship between the chemical properties of the endogenous class A GPCR ligands and the chemical properties of the residue in this position. Most interestingly, the corresponding K296^7.43^ of rhodopsin is covalently linked to retinal, a cofactor with a highly delocalized electronic system [43]. Considering, that rhodopsin is activated upon the interaction between retinal and a single photon, high importance of this delocalized electronic system and the interplay of quantum effects in the initiation of signal transduction could be assumed, at least for this GPCR. In such mechanism K296^7.43^, the first unit of the polar signaling channel, would act as a sensor residue. The high degree of conservation of polar signaling channel residues suggest that this theory could be extended to other class A GPCRs, but indeed, further verification is needed. Analysis of the individual dynamics of these specific side chains revealed that the observed movements are small and mostly occur without the transition between rotameric states (Table S2). Considering that the orientation of amino acid side chains in the orthosteric binding pocket of the MOP are nearly identical in the agonist- [5,6] and antagonist-bound states [7], our results suggest that the underlying event of receptor activation is the parallel change of macroscopic polarization in a shielded central duct of the transmembrane domain. Small, simultaneous changes of side chain orientations of polar signaling channel residues could not necessarily result in direct interactions between them. Small conformational changes of polar or charged residues, however, could result in significant alterations in the local electronic structures, which could then propagate along the structure. The involvement of protons and water molecules in the transmission of signal could also be presumed, since the orthosteric binding pocket and the G protein-binding interface is connected by a hydrated pathway [5,6], and water molecules were observed to exchange rapidly between the internal cavities of the TM domain during simulations (Figure S19, Table S3). Although classical force field methods cannot provide quantitative details of such mechanism, independent mutation data provides direct evidence for the interplay of these polar and charged amino acid side chains during receptor activation. Impaired G protein signaling, or elevation of constitutional activity, was observed for mutant receptors, where residues of the above mentioned polar signaling channel were replaced [12,44,45,46,47,48,49,50,51,52], while receptor activity was preserved in double mutants, where the net charge of channel residues was kept intact [48]. 

### 3.7. TM7 Dipole Moment

**Figure 4:**
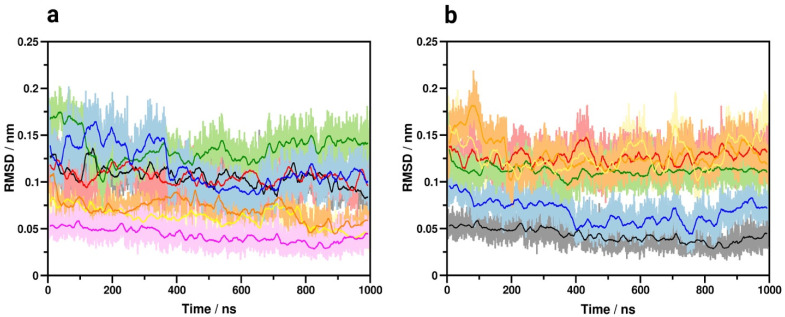
Properties of transmembrane helices. (**a**) Deviation from ideal α-helical geometry in the G_i_ protein-bound active state. Black: TM1, red: TM2, green: TM3, blue: TM4, yellow: TM5, orange: TM6, magenta: TM7. (**b**) Deviation of TM7 from ideal α-helical geometry in the active, G_i_ protein-bound (black), beta-arrestin-2 bound (red), Nb39 nanobody-bound (blue), T4-lysozyme-fused (green), G_i_ protein-bound, ligand free (orange) and G_i_ protein, EM2 and allosteric Na^+^-bound states (yellow).

The shift of macroscopic polarization may be assisted by the inherent dipole moments of TM helices. Generally, a more ordered α-helical segment possesses a higher dipole moment, which can participate in various conduction processes [53]. Further indirect indication of the plausibility of the proposed mechanism is provided by the analysis of the evolution of helix properties during simulations. Results of such analysis revealed that TM7 is the most ordered among the TM helices of the active G_i_ protein-bound receptor. Furthermore, the helicity of TM7 is closest to ideal when the receptor is G_i_ protein-bound, and least ideal when complexed by beta-arrestin-2, presumably providing TM7 with the highest dipole moment in the G_i_ protein-bound state, compared to all other receptor states (Figure 4). This accentuates the role of TM7 in the activation mechanism and it is corroborated by previous reports [12,54]. The role of electrostatic forces and the importance of charge balance is further supported by the known effect of elevated concentrations of Na^+^ ions [9] and the concept of voltage sensing [55,56]. According to this latter, changes in the transmembrane electrostatic potential (V_m_) resulting from the rearrangement of charged species and polar membrane components elicit functional effects in GPCRs.

## 4. Conclusions

The above presented results and considerations, as well as comparison to published mutation data have led us to suggest that large scale structural rearrangements may not be the key event of receptor activation. We suggest that the current theory of GPCR activation could be extended to include that the signal transduction mechanism may be initiated by the perturbation of the electrostatic balance within the binding pocket. Such perturbation is then propagated to the intracellular G protein-binding interface through the minuscule rearrangement of polar amino acid side chains of highly conserved structural motifs, located along TM7, while assisted by the inherent dipole moment of that helical segment. This alternative perspective of the activation mechanism, corroborated by a number of earlier indications [44,45,46,47,48,49,50,51,52], may lead to a more accurate explanation of ligand induced effects in multiple functional states. More importantly, this could highlight certain physico-chemical properties of ligands with different functional properties and may provide a new perspective for medicinal chemists in the pursuit of a new generation of GPCR drugs. 

## Figures and Tables

**Table 1:** Frequency of intramolecular salt bridges and H-bonds expressed as percentages of the total conformational ensemble, generated by MD simulations.

Interactions	Residues Involved			Active State				Inactive State			
		G_i_ Protein Complex, No Ligand	G_i_ Protein Complex, Allosteric Na^+^	G_i_ Protein Complex	Beta-arrestin-2	Nb39 Nanobody	Fused T4-lysozyme	G_i_ Protein Complex	Beta-arrestin-2	Nb39 Nanobody	Fused T4-lysozyme
**Salt bridges**											
DRY-H8	R165^3.50^; D340^8.47^	6.9	34.12	53.5	8.8	29.4	53.0	0.0	0.0	0.0	0.0
intra-DRY	D164^3.49^; R165^3.50^	9.1	0.96	0.1	5.4	0.1	0.3	8.9	10.9	17.9	6.4
**H-bonds**											
intra-DRY	D164^3.49^; R165^3.50^	63.7	1.25	0.2	19.3	0.0	0.9	21.7	35.1	82.6	11.0
DRY-ICL2	D164^3.49^; R179	0.4	99.0	98.1	99.6	100.0	100.0	100.0	100.0	100.0	95.2
DRY-TM5	R165^3.50^; Y252^5.58^	0.0	0.2	0.0	5.7	5.7	3.1	0.0	0.0	0.0	0.0
DRY-TM6	R165^3.50^; T279^6.34^	0.0	0.0	0.0	0.0	0.0	0.0	37.7	2.4	98.8	91.0
CWxP-TM7	C292^6.47^-W293^6.48^; N328^7.45^	64.5	0.2	18.4	0.8	7.3	33.3	0.0	4.6	0.1	0.7
NPxxY-TM network ^†^	N332^7.49^, Y336^7.53^; L158^3.43^, Y252^5.58^, V285^6.40^	0.6	44.4	5.5	3.4	40.7	7.1	0.3	0	0.1	0.0

BP = orthosteric binding pocket of the mu opioid receptor; EM2 = endomorphin-2; ICL2 = 2nd intracellular loop of the mu opioid receptor; H5 = helix 5 of the G_i_ protein α subunit; FL / ML / CL = finger loop / middle loop / C loop of beta-arrestin-2; Nb39 = Nb39 nanobody. Ballesteros-Weinstein numbering of residues is indicated in superscript. ^†^ Described in reference [5].

## Supplementary Materials

The following are available online at https://www.mdpi.com/article/10.3390/biom11050670/s1, Figure S1: Comparison of the constructed peptide agonist-bound active MOP-G_i_ protein to the recently published cryo-electron microscopic structure, Figure S2: Analysis data for a representative folding simulation of the N-terminal domain of the MOP, Figures S3 and S4: Analysis of the dynamics of N- and C-terminal domains, Figure S5: Analysis of membrane properties during equilibration and production simulations, Figures S6 and S7: Analysis of Na^+^ ion presence in the allosteric and orthosteric binding sites, Figure S8: TM6 disposition analysis, Figure S9: Secondary structure of ICL1, Figure S10: Secondary structure of H8, Figure S11: Secondary structure of ICL2, Figure S12: Intermolecular H-bonds between ICL2 and the G_i_ protein, Figures S13–S17: Dynamic cross-correlation matrices, Figure S18: The polar signaling channel of the G_i_ protein or beta-arrestin-2-bound MOP, Figure S19: Transmembrane water network in the active, G_i_ protein-bound MOP, Table S1: Intermolecular salt bridges and H-bonds, Table S2: Side chain rotameric states and transitions of the residues of the polar signaling channel, Table S3: Water occupancy in the transmembrane domain of the active, G_i_ protein-bound MOP.
