# Supplementary material for: Correlated Motions of Conserved Polar Motifs Lay out a Plausible Mechanism of G Protein-Coupled Receptor Activation
Source: Biomolecules. 2021 Apr 30;11(5):670. doi: 10.3390/biom11050670 (PMC8146931; doi:10.3390/biom11050670)
Supplement: Supplementary file 1 [file biomolecules-11-00670-s001.zip › biomolecules-1174999-supplementary.pdf]

Supporting Information for

Correlated motions of conserved polar motifs lay out a plausible mechanism of G protein-coupled receptor activation.

Argha Mitra, Arijit Sarkar, Márton R. Szabó†, Attila Borics.*

Laboratory of Chemical Biology, Institute of Biochemistry, Biological Research Centre, Szeged, 62. Temesvári krt., Szeged, Hungary, H-6726.

***Corresponding Author**

Email: borics.attila@brc.hu, Phone: +36 62 599 600 ext. 430.

† Present Address:

Department of Biochemistry, Faculty of Medicine, University of Szeged, 9 Dóm sq. Szeged, Hungary, H-6720

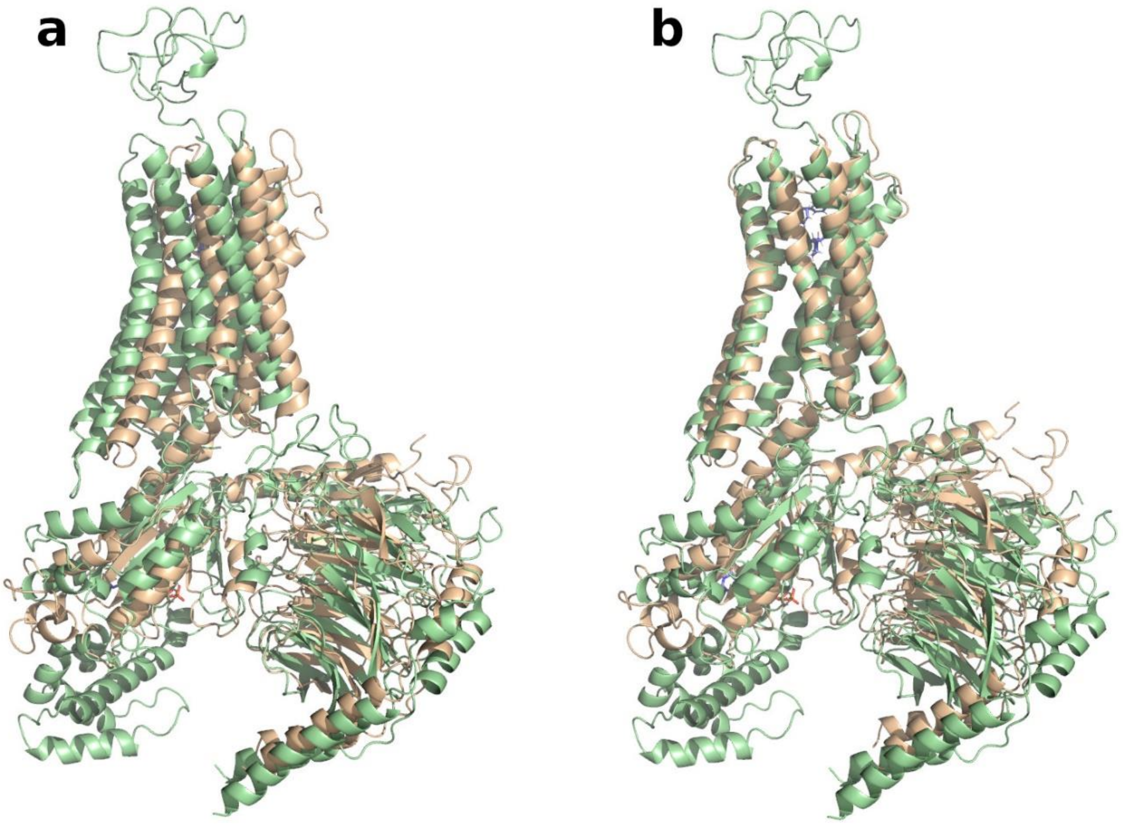

Figure S1. Comparison of the constructed peptide agonist-bound active μ-opioid receptor (MOP) – G_i protein complex (green) to the recently published cryo-electron microscopic structure (brown, pdb code: 6DDE), by (a) fitting of all C_α atoms (RMSD = 0.623 nm), or (b) fitting only the C_α atoms of the transmembrane domains (RMSD = 0.189 nm).

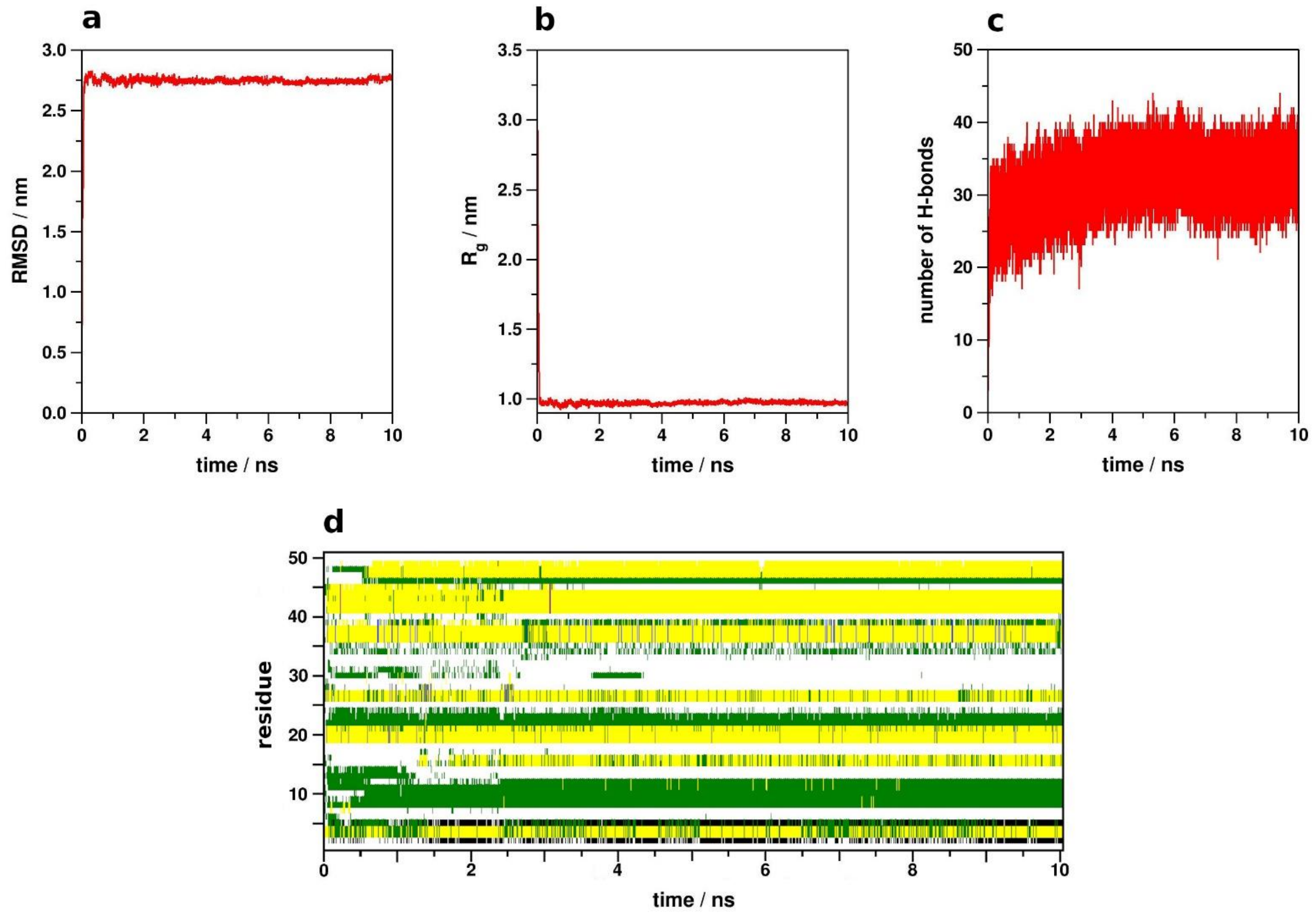

Figure S2. Analysis data for a representative folding simulation of the N-terminal domain of the MOP. (a) Backbone RMSD with respect to the fully extended starting structure. (b) The evolution of the radius of gyration. (c) The number of intramolecular H-bonds formed during the simulation. (d) DSSP analysis of the evolution of secondary structure during the simulation. (white: unordered; green: bend; yellow: β-turn; black: β-bridge, grey: 3_{10} helix; blue: α-helix)

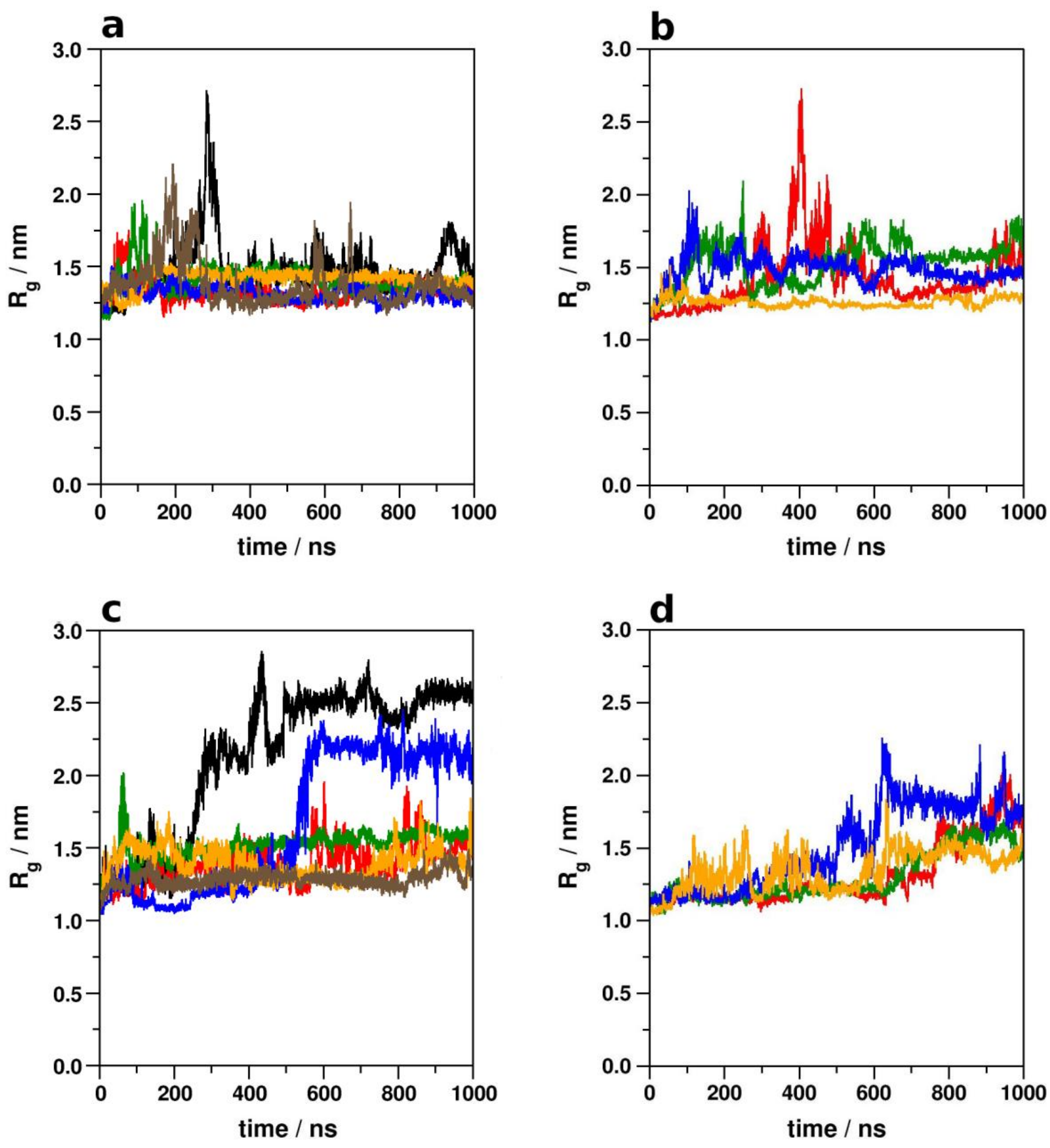

Figure S3. The evolution of radii of gyration of the N- (a, b) and C-terminal domains (c, d) of the active (a, c) and inactive state (b, d) MOP during production simulations. Black: MOP – G_i protein complex, without ligand; brown: MOP - G_i protein complex with bound allosteric Na^+; red: MOP – G_i protein complex; green: MOP - beta-arrestin-2 complex; blue: MOP – Nb39 nanobody complex; orange: MOP – T4-lysozyme fusion.

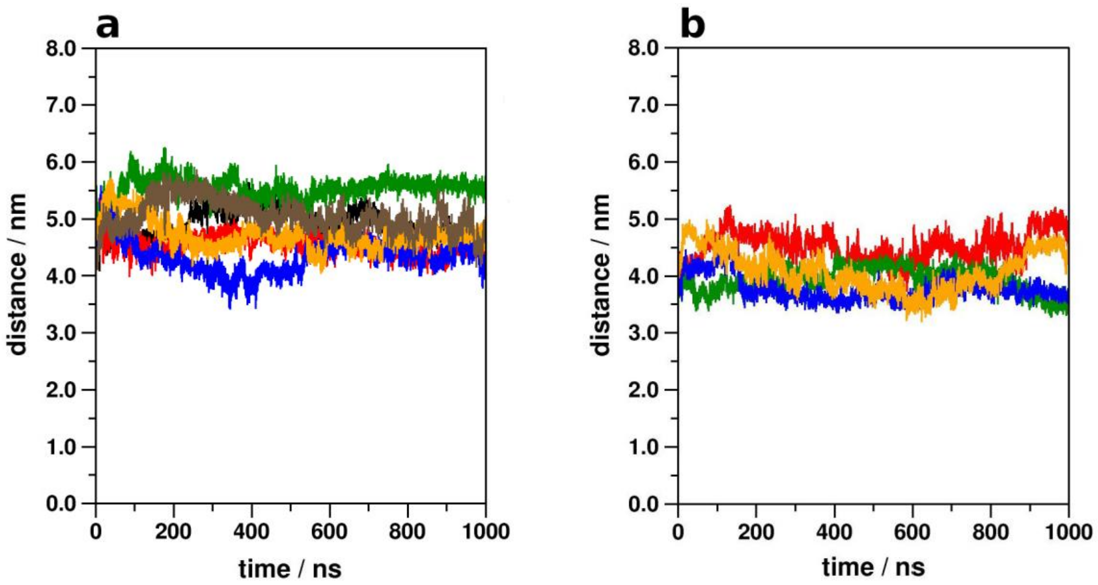

Figure S4. Minimum distance between the N- and C-terminal domains (and their periodic replicas) of the active (a) and inactive state (b) MOP during production simulations. Black: MOP – G_i protein complex, without ligand; brown: MOP - G_i protein complex with bound allosteric Na^+; red: MOP – G_i protein complex; green: MOP – beta-arrestin-2 complex; blue: MOP – Nb39 nanobody complex; orange: MOP – T4-lysozyme fusion.

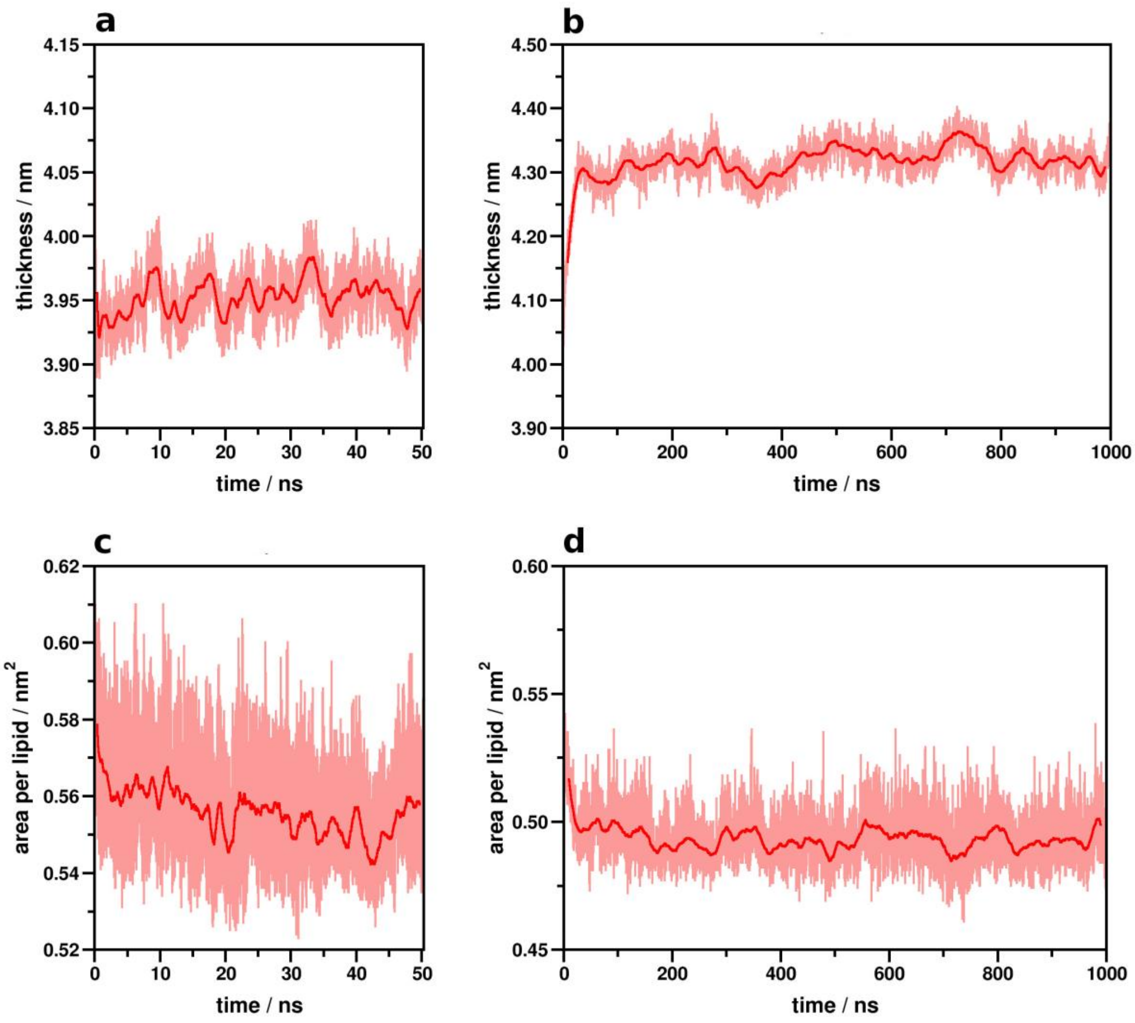

Figure S5. Analysis of membrane properties during equilibration and production simulation of the EM2-bound active MOP – G_i protein complex as a representative example. (a) Membrane bilayer thickness in the equilibrium phase. (b) Membrane bilayer thickness in the production phase. (c) Area per lipid headgroup in the equilibration phase. (d) Area per lipid headgroup in the production phase.

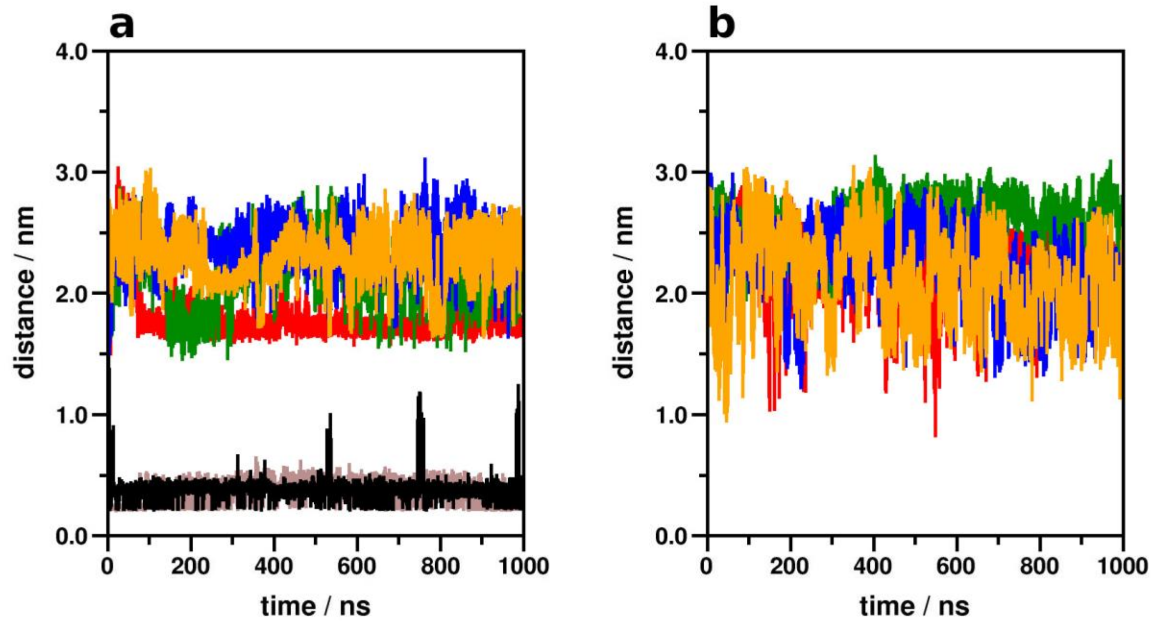

Figure S6. Minimum distance between Na^+ ions and the allosteric Na^+ binding site, D114$^{2.50}$ of the active (a) and inactive state (b) MOP during simulations. Black: MOP – G_i protein complex, without ligand; brown: MOP - G_i protein complex with bound allosteric Na^+; red: MOP – G_i protein complex; green: MOP – beta-arrestin-2 complex; blue: MOP – Nb39 nanobody complex; orange: MOP – T4-lysozyme fusion.

a

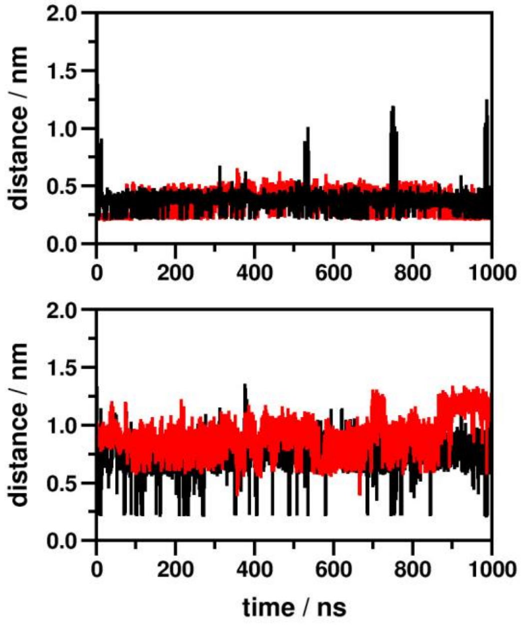

b

residue	frequency of contact / %	
	ligand-free	EM2 + Na^+
D114$^{2.50}$	72.90	50.90
D147$^{3.32}$	0.92	0.01
S154$^{3.39}$	93.91	76.58
W293$^{6.48}$	40.50	19.16
N328$^{7.45}$	3.88	59.42
S329$^{7.46}$	11.61	32.46
N332$^{7.49}$	5.24	37.54
Y336$^{7.53}$	0.66	11.32

Figure S7. (a) Minimum distance between Na^+ ions and the allosteric and orthosteric binding sites, D114$^{2.50}$ (top panel) and D147$^{3.32}$ (bottom panel), respectively, of the active state ligand-free MOP (black) and the MOP with bound by EM2 and allosteric Na^+ (red) during simulations. (b) The frequency of contact ($d \leq 0.4$ nm) between Na^+ ions and polar amino acid side chains of the allosteric and orthosteric binding pockets.

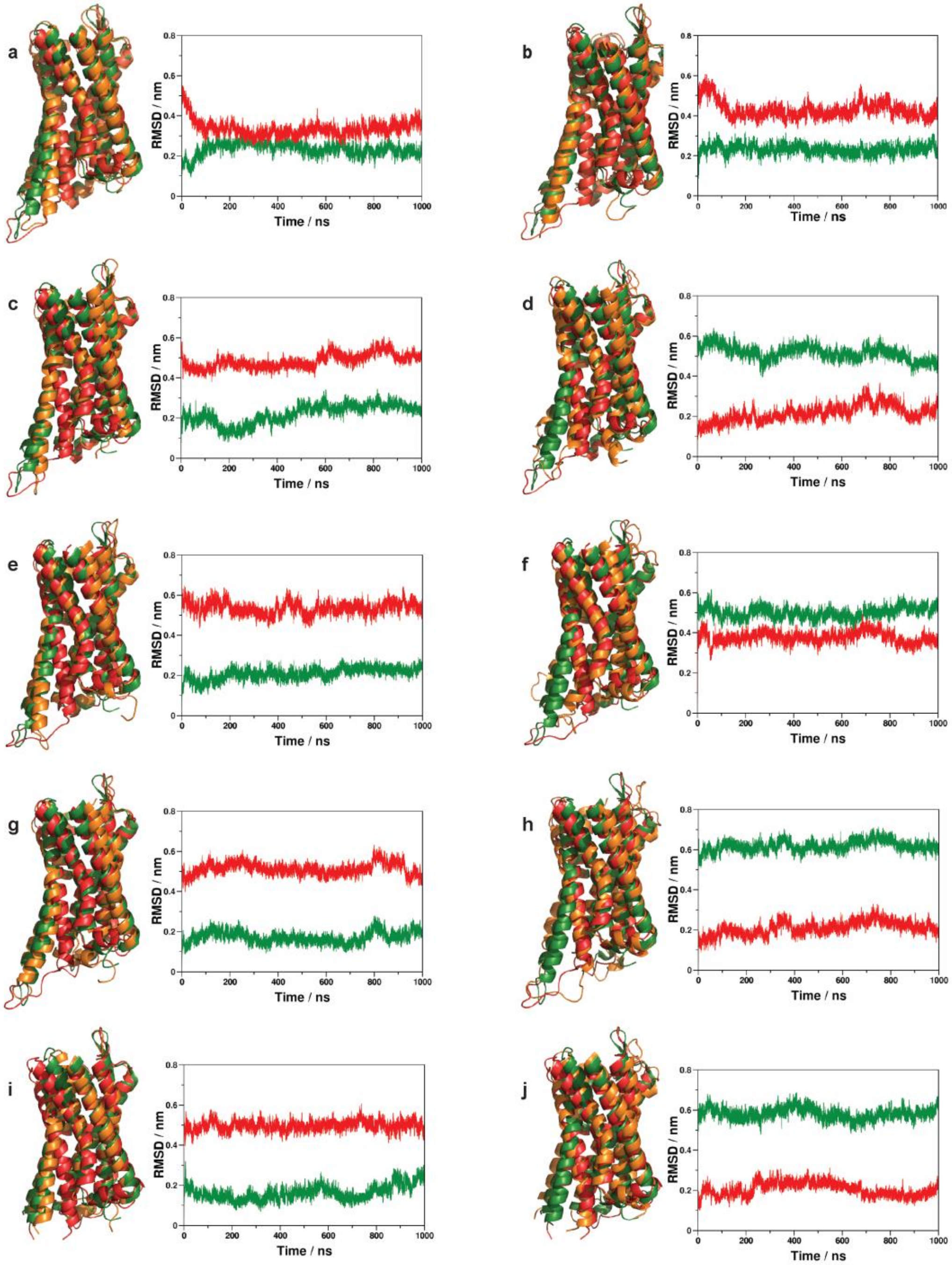

Figure S8. Disposition of TM6 during simulations (orange) with respect to the active (green) and inactive (red) crystallographic structures of the MOP. (a) Active receptor – G_i protein complex, without ligand. (b) Active receptor - G_i protein complex with bound allosteric Na^+. (c) Active receptor – G_i protein complex. (d) Inactive receptor – G_i protein complex. (e) Active receptor – beta-arrestin-2 complex. (f) Inactive receptor – beta-arrestin-2 complex. (g) Active receptor – Nb39 nanobody complex. (h) Inactive receptor – Nb39 nanobody complex. (i) Active receptor – T4-lysozyme fusion. (j) Inactive receptor – T4-lysozyme fusion.

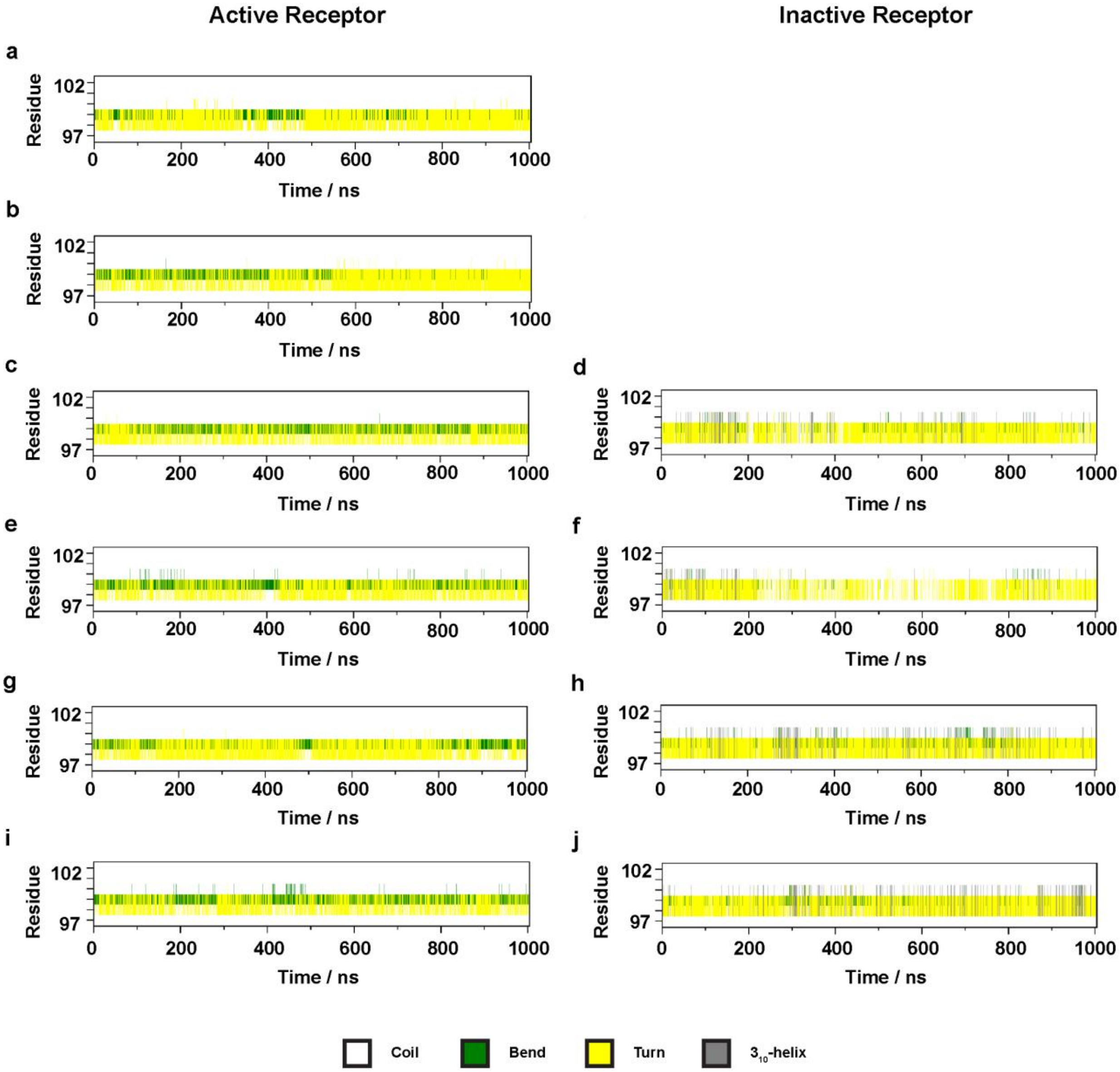

Figure S9. Evolution of the secondary structure of ICL1 during simulations. (a) Active receptor – G_i protein complex, without ligand. (b) Active receptor - G_i protein complex with bound allosteric Na^+. (c) Active receptor – G_i protein complex. (d) Inactive receptor – G_i protein complex. (e) Active receptor – beta-arrestin-2 complex. (f) Inactive receptor – beta-arrestin-2 complex. (g) Active receptor – Nb39 nanobody complex. (h) Inactive receptor – Nb39 nanobody complex. (i) Active receptor – T4-lysozyme fusion. (j) Inactive receptor – T4-lysozyme fusion.

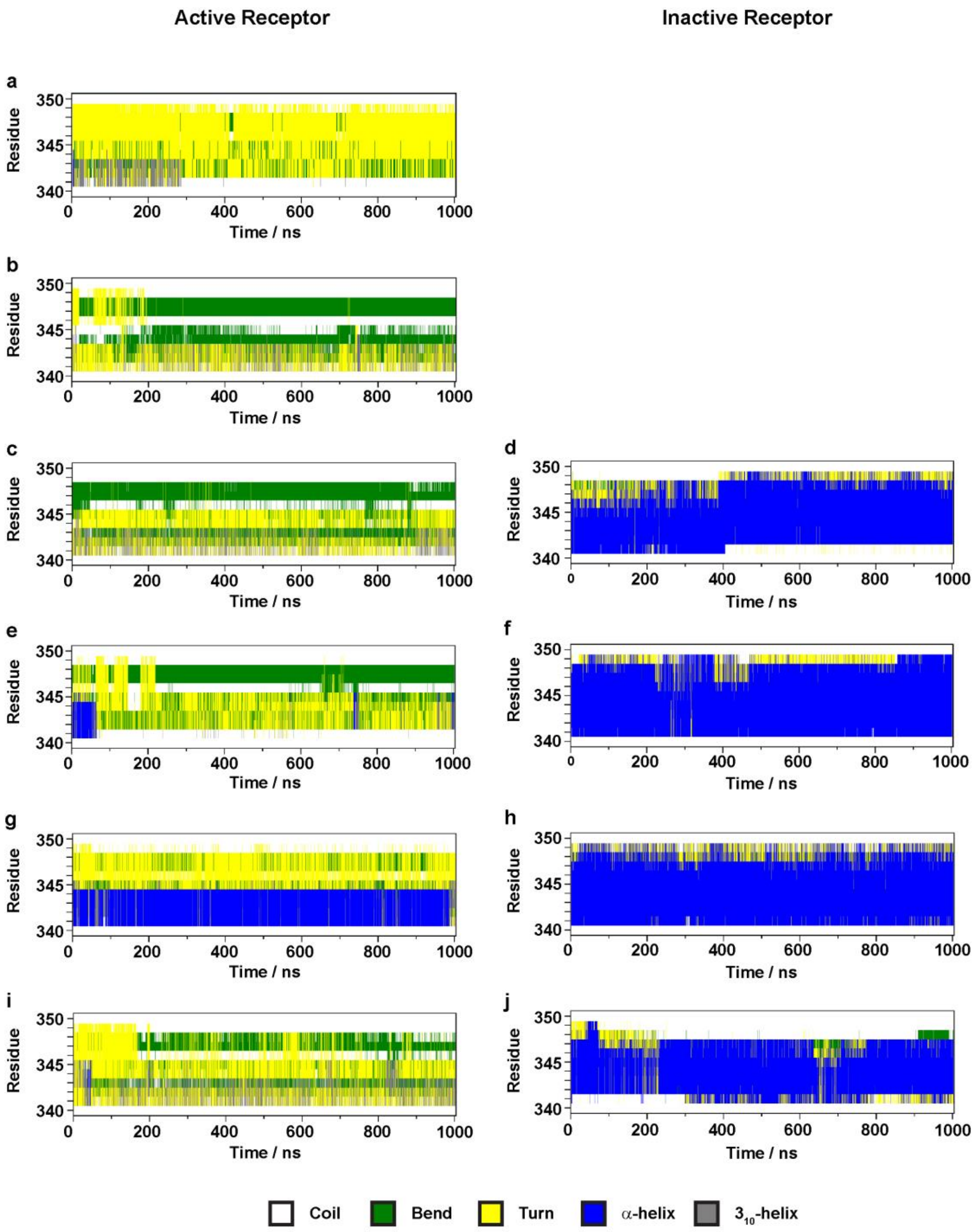

Figure S10. Evolution of the secondary structure of H8 during simulations. (a) Active receptor – G_i protein complex, without ligand (b) Active receptor – G_i protein complex with bound allosteric Na^+. (c) Active receptor - G_i protein complex. (d) Inactive receptor – G_i protein complex. (e) Active receptor – beta-arrestin-2 complex. (f) Inactive receptor – beta-arrestin-2 complex. (g) Active receptor – Nb39 nanobody complex. (h) Inactive receptor – Nb39 nanobody complex. (i) Active receptor – T4-lysozyme fusion. (j) Inactive receptor – T4-lysozyme fusion.

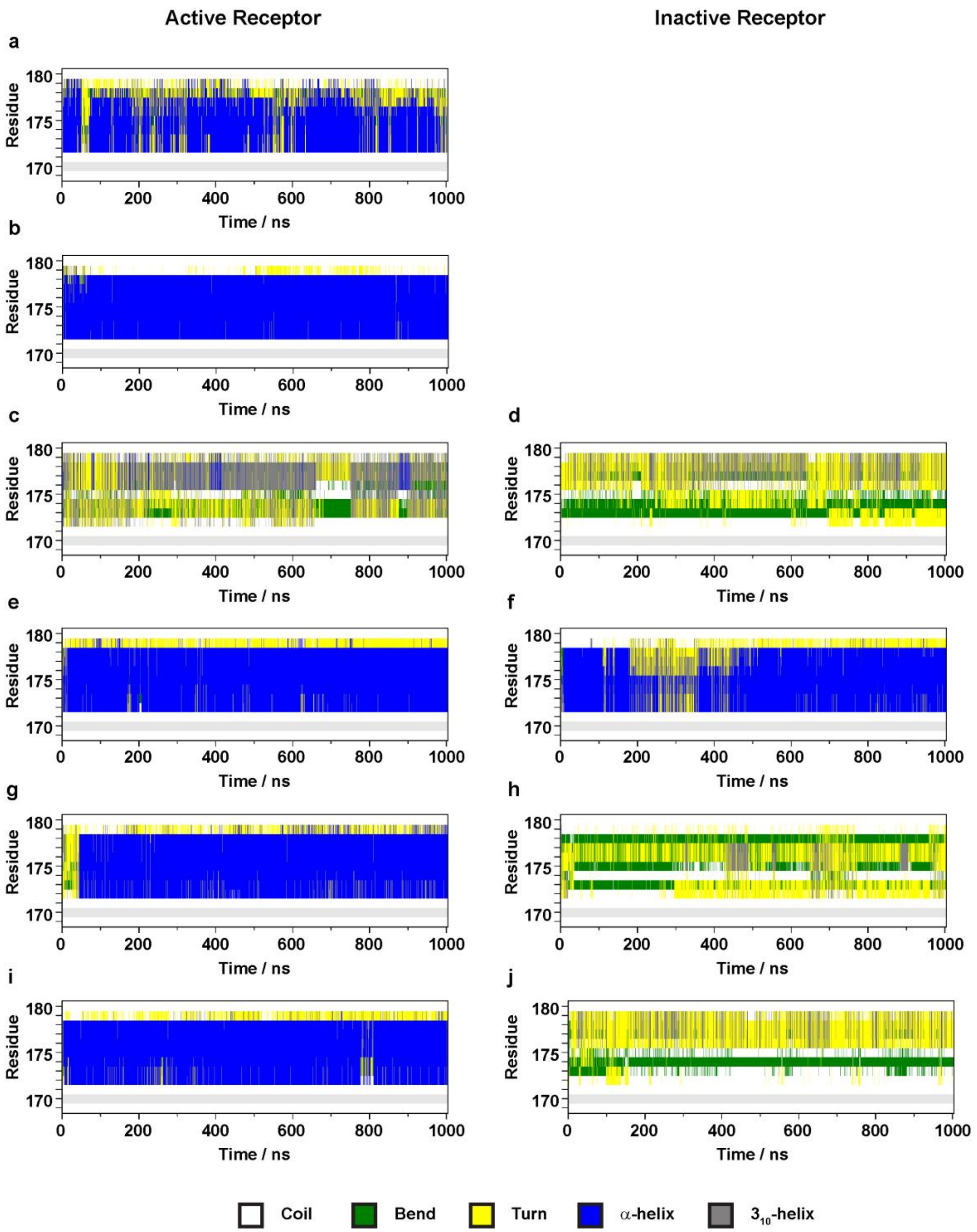

Figure S11. Evolution of the secondary structure of ICL2 during simulations. (a) Active receptor – G_i protein complex, without ligand (b) Active receptor - G_i protein complex with bound allosteric Na^+. (c) Active receptor – G_i protein complex. (d) Inactive receptor – G_i protein complex. (e) Active receptor – beta-arrestin-2 complex. (f) Inactive receptor – beta-arrestin-2 complex. (g) Active receptor – Nb39 nanobody complex. (h) Inactive receptor – Nb39 nanobody complex. (i) Active receptor – T4-lysozyme fusion. (j) Inactive receptor – T4-lysozyme fusion.

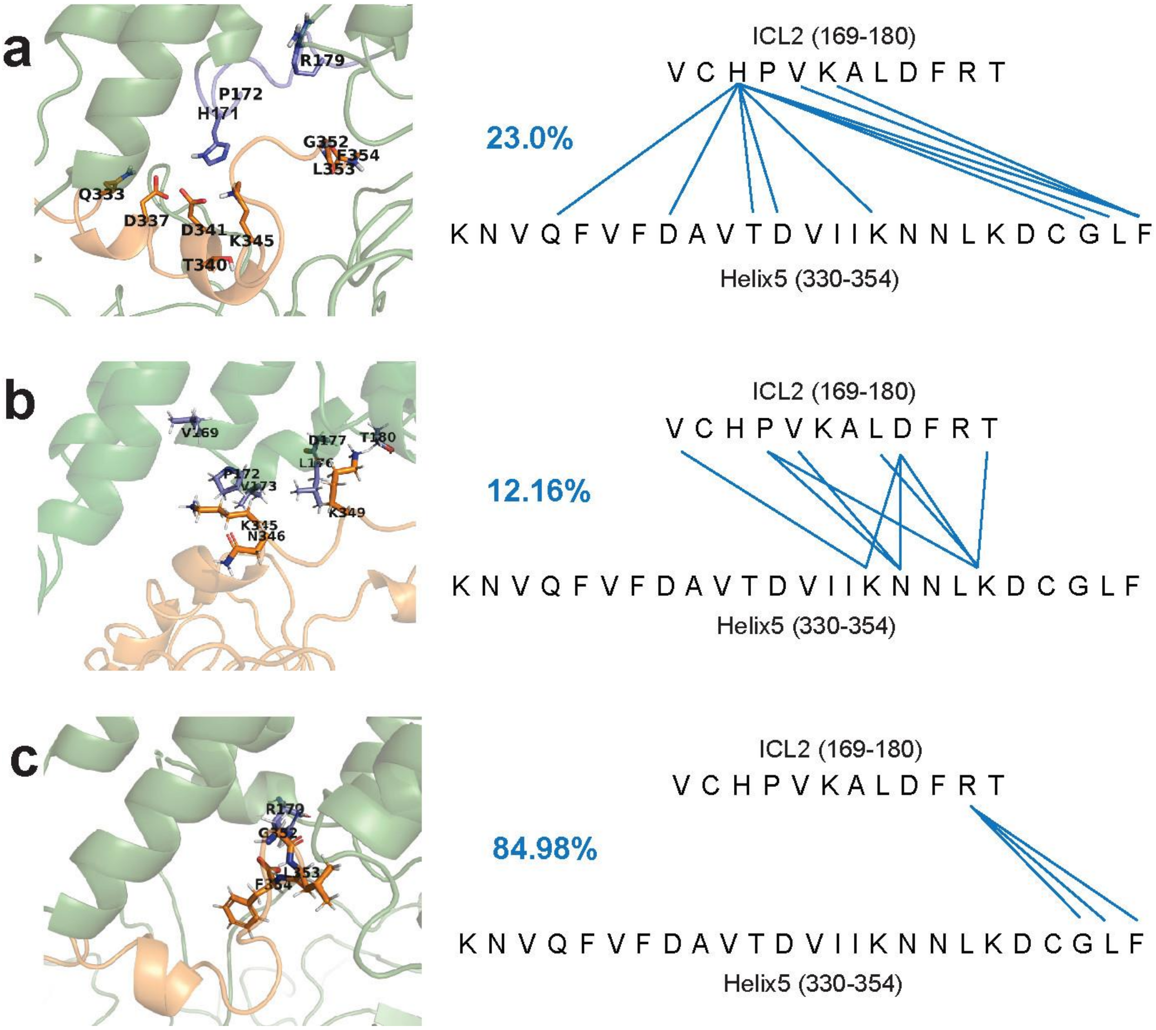

Figure S12. Frequency and donor and acceptor sites of intermolecular H-bonds between ICL2 of the MOP and the G_i protein. Frequency of H-bonds are expressed as percentages of the total structural ensemble and indicated by blue numbers. ICL2 is shown as blue cartoon and sticks. Helix 5 of the $G_i\alpha$ subunit is shown as orange cartoon and sticks. (a) EM2-bound active receptor and the $G_i\alpha$ subunit. (b) EM2-bound active receptor and the $G_i\alpha$ subunit, in the presence of allosteric Na^+. (c) Active receptor and the $G_i\alpha$ subunit in the absence of EM2.

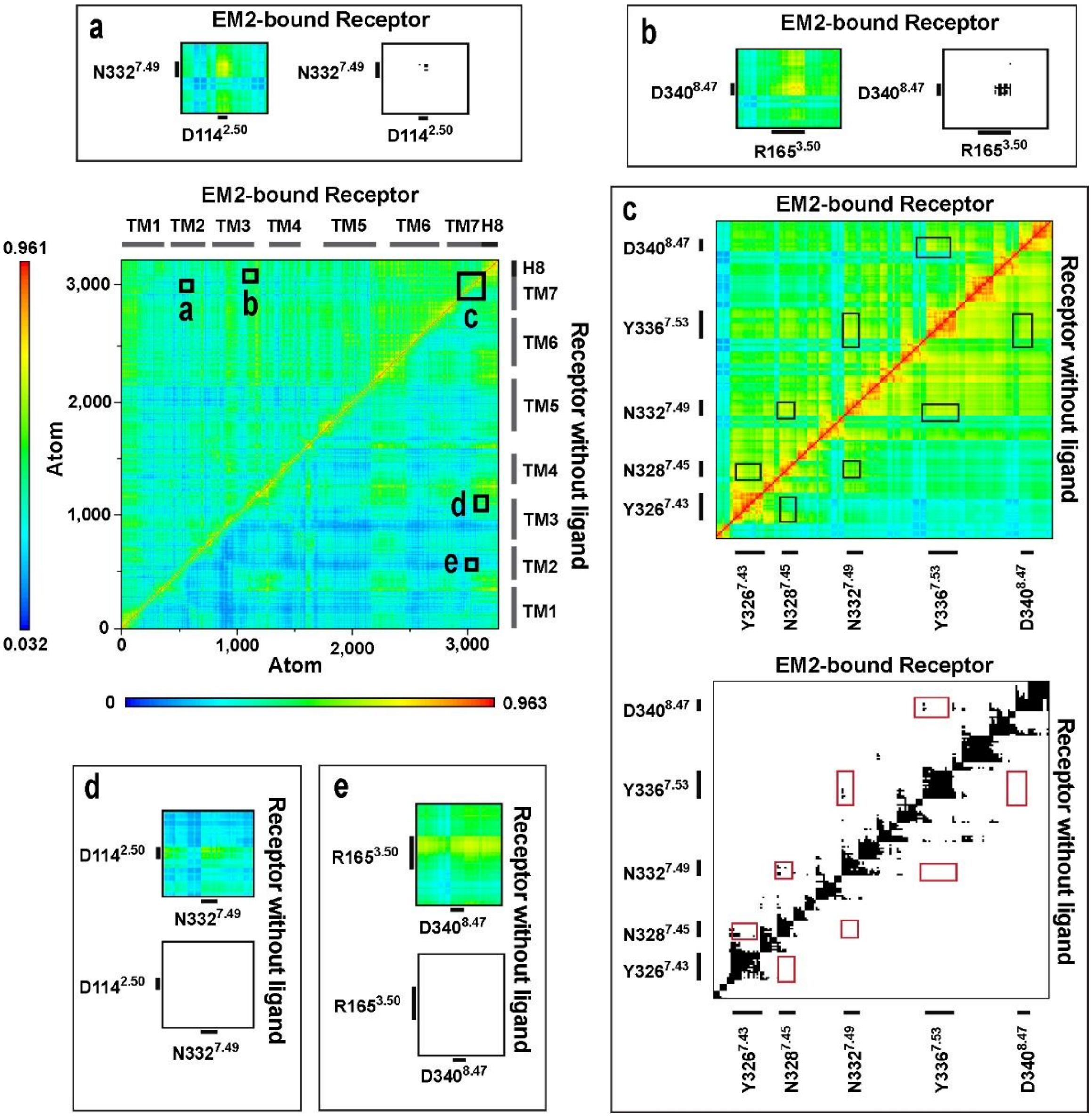

Figure S13. Dynamic cross-correlation matrices of the G_i protein-bound MOP in the active state in absence and presence of orthosterically bound peptide agonist EM2. Panels (a-e) are magnified views of regions of amino acid residues of interest. Black and white panels show correlations above the threshold of 0.7 MI.

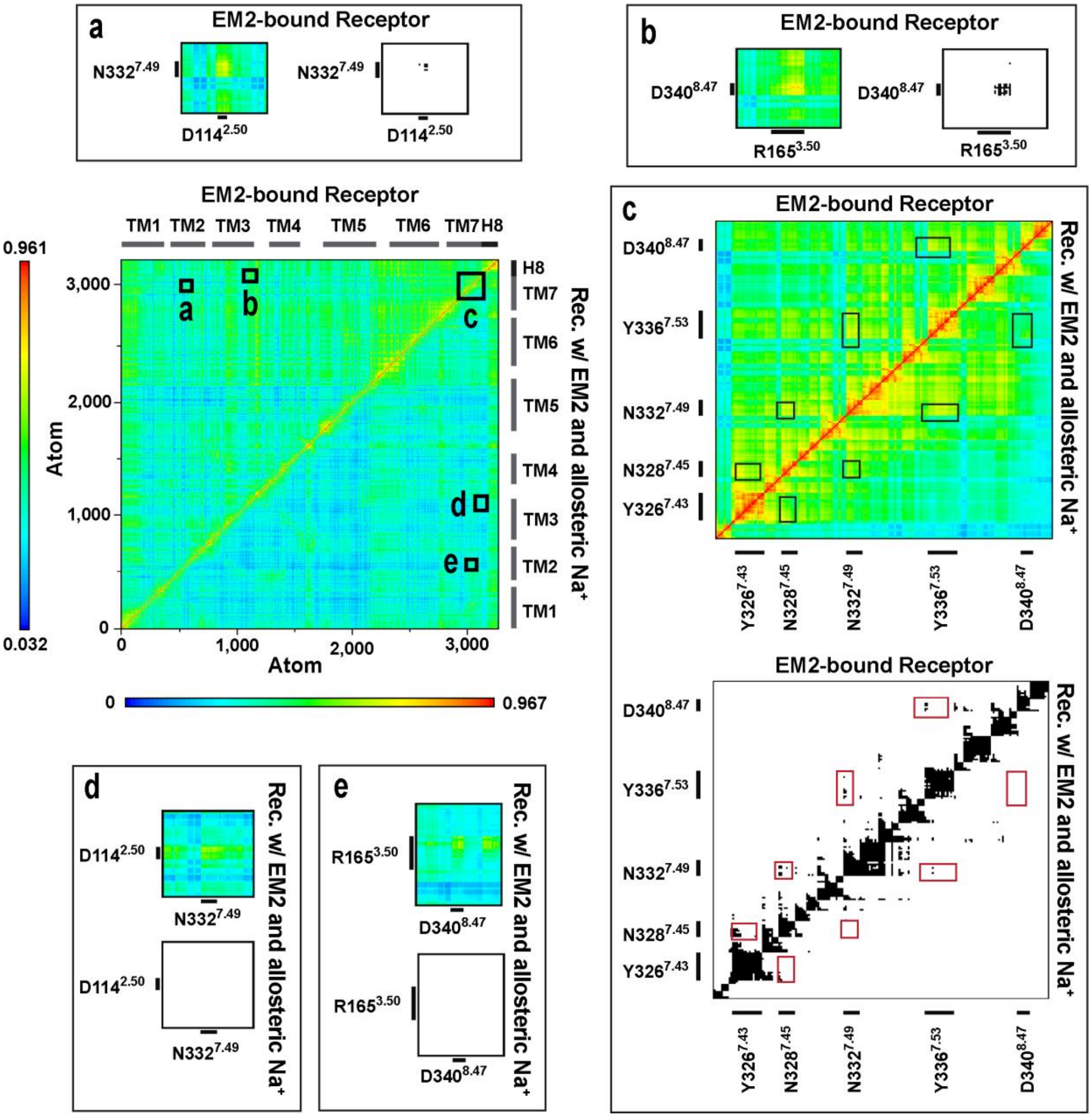

Figure S14. Dynamic cross-correlation matrices of the G_i protein and EM2-bound MOP in the active state in absence and presence of an allosterically bound Na^+ ion. Panels (a-e) are magnified views of regions of amino acid residues of interest. Black and white panels show correlations above the threshold of 0.7 MI.

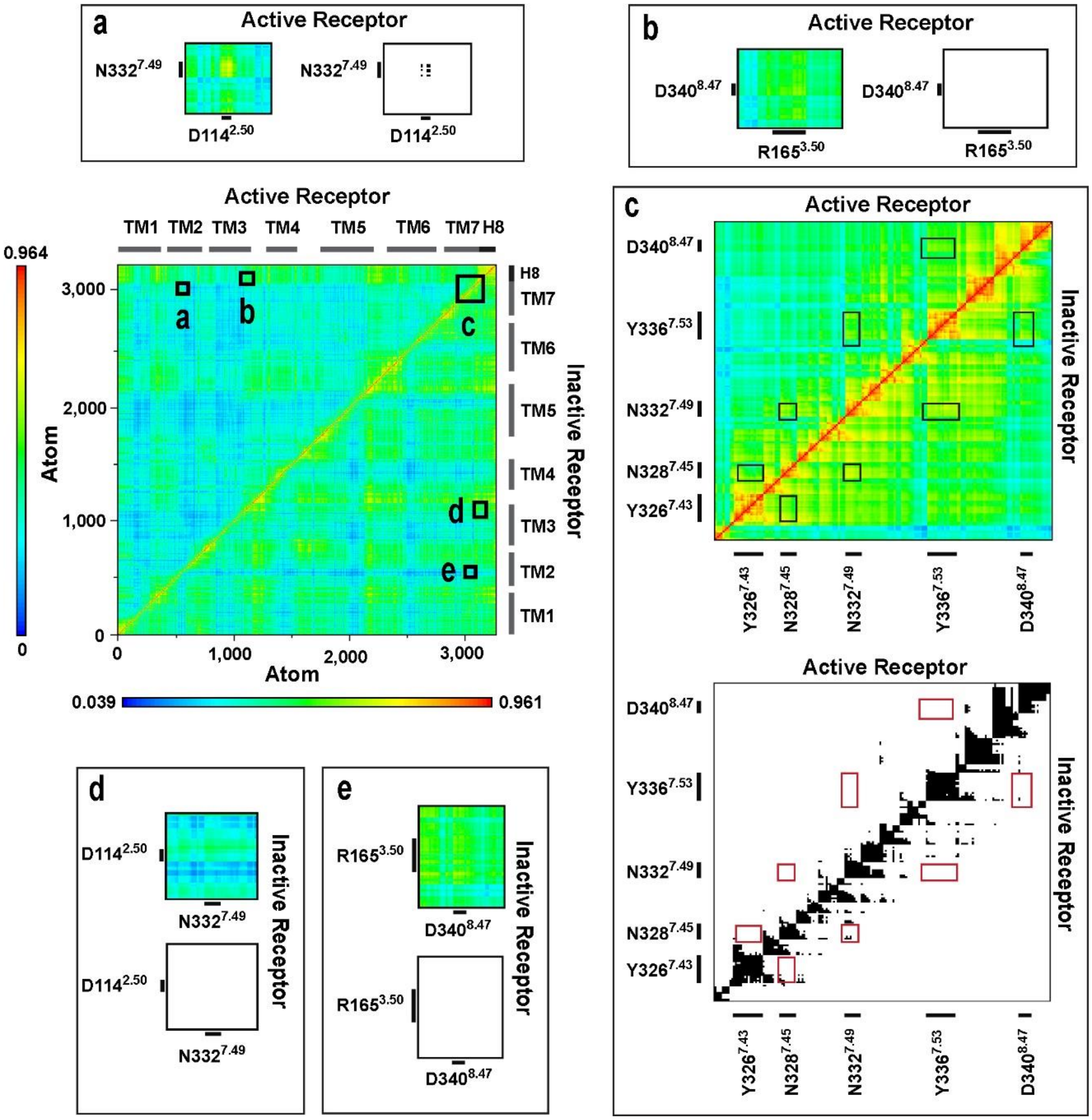

Figure S15. Dynamic cross-correlation matrices of the beta-arrestin-2-bound MOP in active and inactive states. Panels (a-e) are magnified views of regions of amino acid residues of interest. Black and white panels show correlations above the threshold of 0.7 MI.

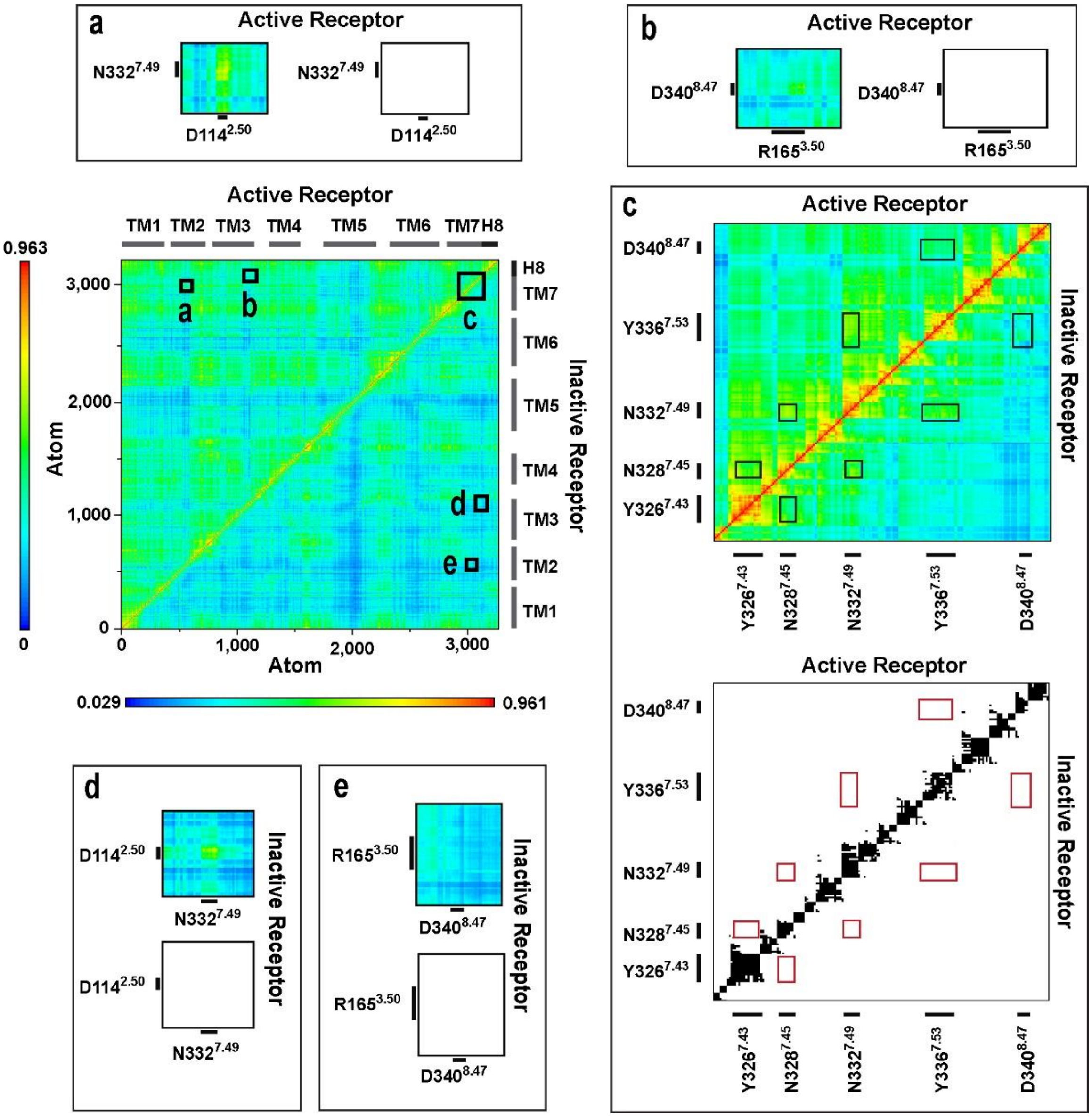

Figure S16. Dynamic cross-correlation matrices of the Nb39 nanobody-bound MOP in active and inactive states. Panels (a-e) are magnified views of regions of amino acid residues of interest. Black and white panels show correlations above the threshold of 0.7 MI.

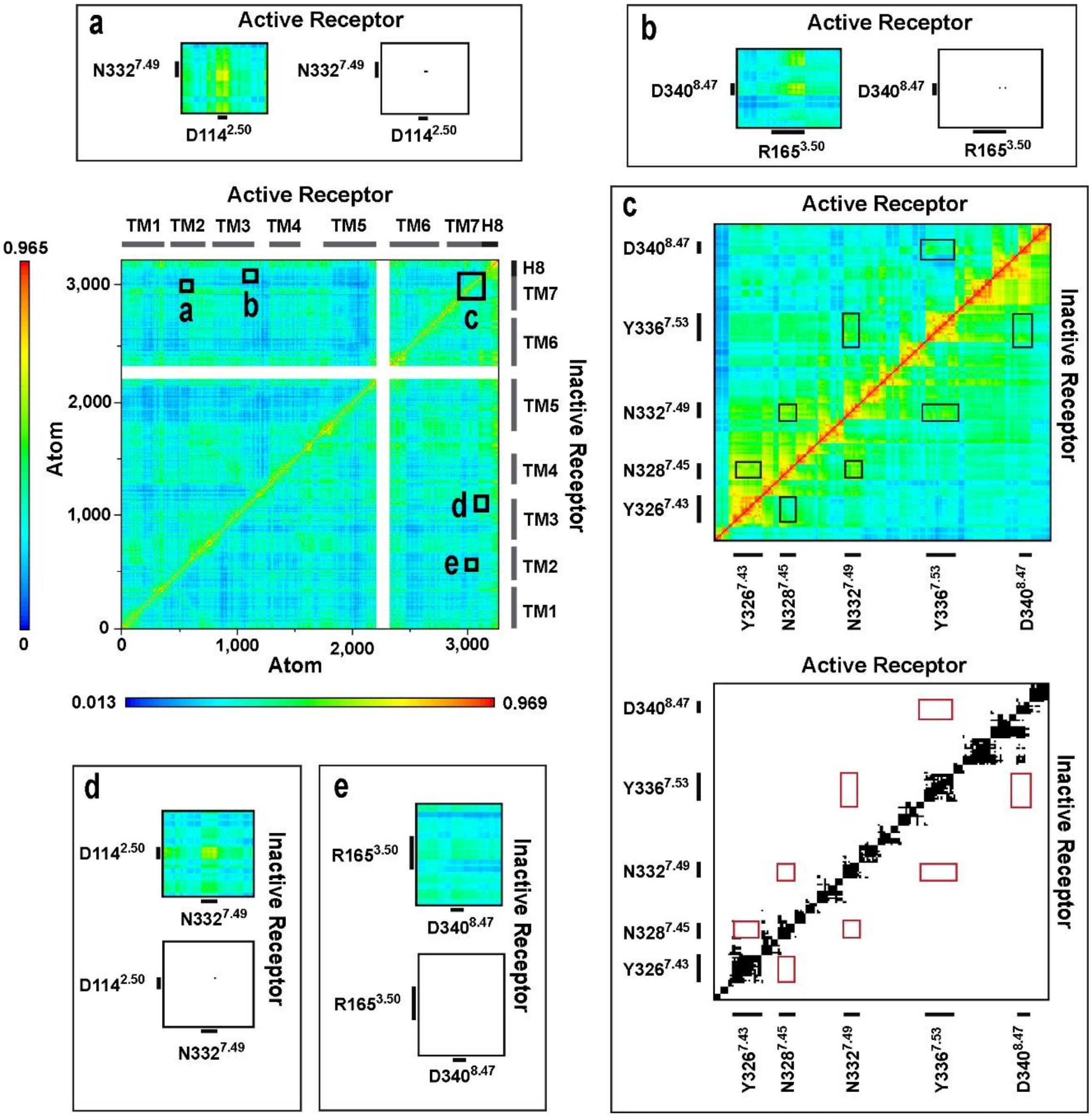

Figure S17. Dynamic cross-correlation matrices of the active and inactive MOP-T4-lysozyme fusion proteins. Panels (a-e) are magnified views of regions of amino acid residues of interest. Black and white panels show correlations above the threshold of 0.7 MI.

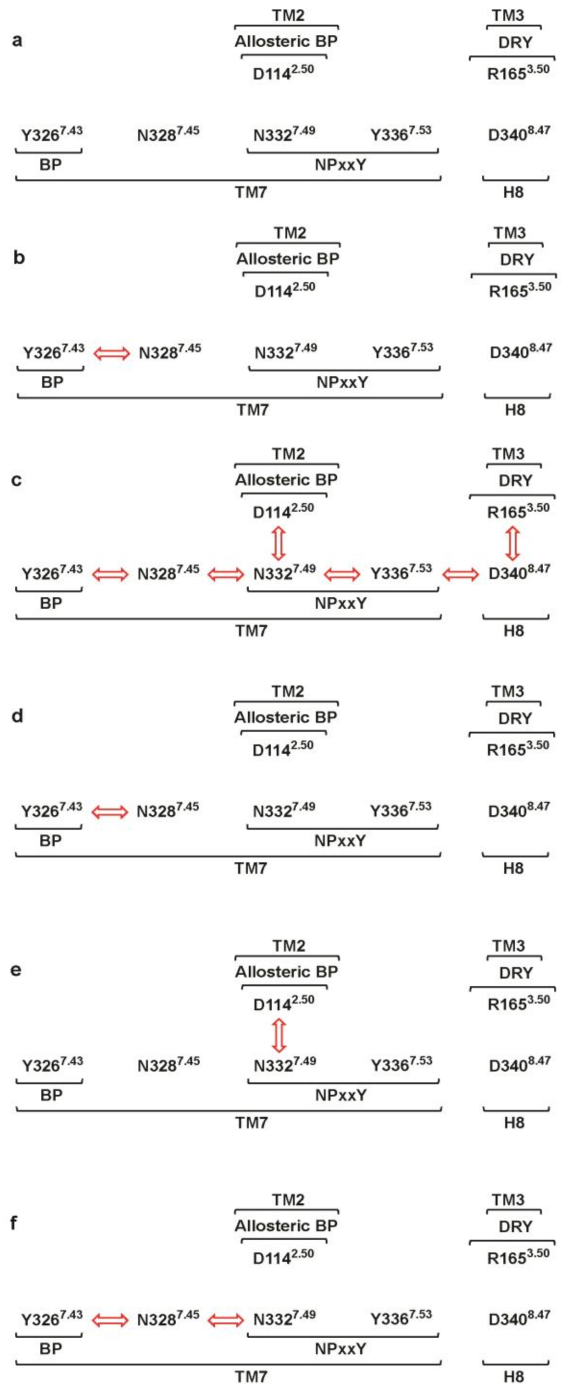

Figure S18. The polar signaling channel of the G_i protein or beta-arrestin-2-bound MOP revealed by dynamic cross correlation analysis. Red arrows indicate correlated motions of the respective amino acids in the (a) active receptor – G_i protein, without EM2, (b) active receptor - G_i protein complex with bound allosteric Na^+. (c) active receptor – G_i protein, (d) inactive receptor – G_i protein, (e) active receptor – beta-arrestin-2 and (f) inactive receptor – beta-arrestin-2 complexes.

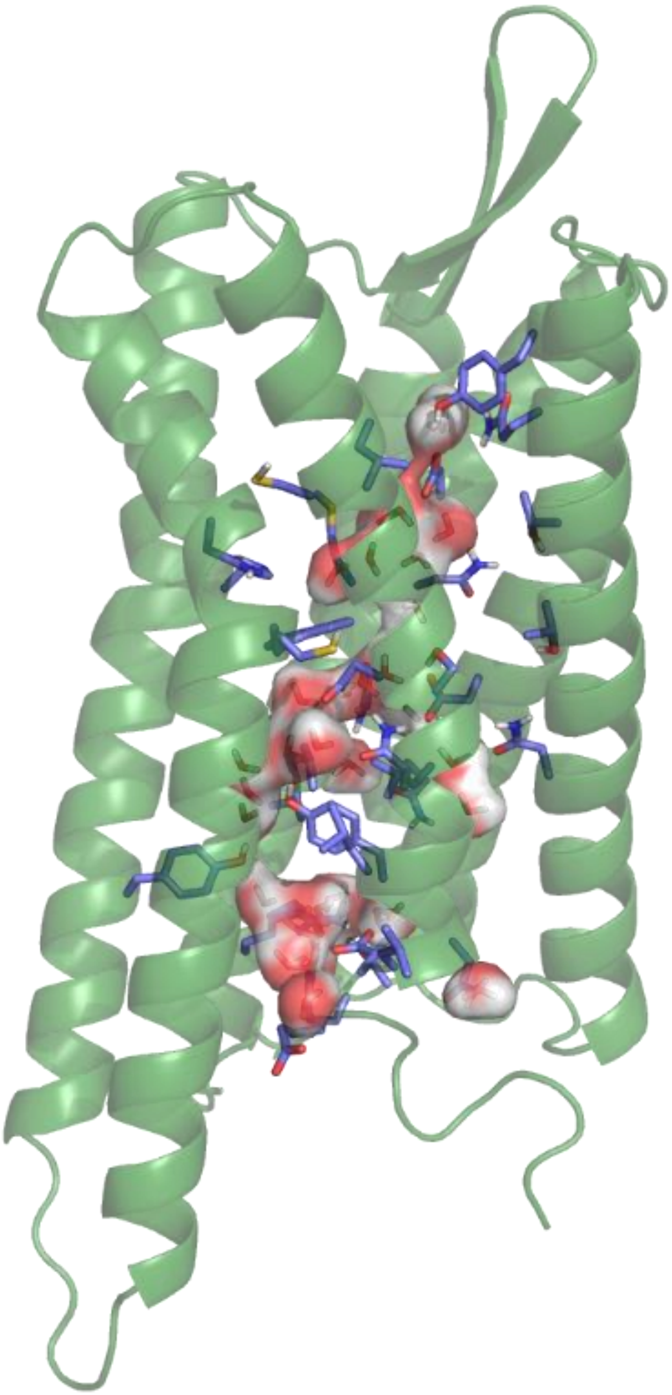

Figure S19. Snapshot of the transmembrane domain of the active, EM2-bound, G protein-coupled MOP. Residues in contact with water molecules during the course of simulation are shown in stick representation. Water molecules are shown in surface representation.

Table S1. Frequency of intermolecular salt bridges and H-bonds expressed as percentages of the total conformational ensemble, generated by MD simulations.

	Residues involved, respectively	Active state						Inactive state			
		G_i protein complex, no ligand	G_i protein complex, allosteric Na^+	G_i protein complex	Beta-arrestin-2	Nb39 nanobody	Fused T4-lysozyme	G_i protein complex	Beta-arrestin-2	Nb39 nanobody	Fused T4-lysozyme
**Salt bridges**											
BP - EM2	$D147^{3.32}$; Y1	-	76.15	68.4	76.2	60.0	59.2	11.7	3.6	7.0	1.3
**H-bonds**											
BP - EM2	$D147^{3.32}$; Y1	-	99.08	97.0	98.8	86.2	98.1	18.4	15.0	13.4	1.7
ICL2 - H5	V169-T180; K330-F354	84.5	12.16	23.0	-	-	-	3.8	-	-	-
ICL2 - FL	V169-T180; G65-K78	-	-	-	40.8	-	-	-	1.4	-	-
ICL2 - ML	V169-T180; P132-A140	-	-	-	86.4	-	-	-	87.3	-	-
ICL2 - CL	V169-T180; L244-S246	-	-	-	19.3	-	-	-	0.0	-	-
ICL2 - Nb39	V169-T180; T52-V74	-	-	-	-	98.7	-	-	-	16.3	-

BP = orthosteric binding pocket of the MOP; EM2 = endomorphin-2; ICL2 = 2nd intracellular loop of the MOP; H5 = helix 5 of the G_i protein α subunit; FL / ML / CL = finger loop / middle loop / C loop of beta-arrestin-2; Nb39 = Nb39 nanobody. Ballesteros-Weinstein numbering of residues is indicated in superscript.

Table S2. Side chain rotameric states and transitions of the residues of the polar signaling channel.

with the G_i protein complex and without ligand	active				
	rotamer				
residue	g+	g-	t	S^2	tr/ns†
D114$^{2.50}$		1.00		0.99	0.00
R165$^{3.50}$		0.99	0.01	0.95	0.10
Y326$^{7.43}$		1.00		0.98	0.00
N328$^{7.45}$			1.00	0.96	0.10
N332$^{7.49}$		0.81	0.19	0.56	0.04
Y336$^{7.53}$		1.00		0.98	0.00
D340$^{8.47}$			1.00	0.97	0.04
I155$^{3.40}$	0.17	0.79	0.04	0.43	0.09
F289$^{6.44}$			1.00	0.97	0.01
W293$^{6.48}$		1.00		0.98	0.00

with the G_i protein and allosteric Na^+	active				
	rotamer				
residue	g+	g-	t	S^2	tr/ns†
D114$^{2.50}$		1.00		0.98	0.00
R165$^{3.50}$		1.00		0.98	0.00
Y326$^{7.43}$		1.00		0.98	0.00
N328$^{7.45}$	0.01	0.01	0.98	0.92	0.07
N332$^{7.49}$		0.27	0.73	0.48	0.12
Y336$^{7.53}$		1.00		0.97	0.00
D340$^{8.47}$			1.00	0.98	0.00
I155$^{3.40}$	0.36	0.05	0.59	0.14	0.37
F289$^{6.44}$			1.00	0.97	0.00
W293$^{6.48}$			1.00	0.97	0.00

	with the G_i protein complex									
	active					inactive				
	rotamer					rotamer				
residue	g+	g-	t	S^2	tr/ns†	g+	g-	t	S^2	tr/ns†
D114$^{2.50}$		1.00		0.98	0.00		1.00		0.98	0.05
R165$^{3.50}$		0.98	0.02	0.95	0.05		0.09	0.91	0.86	6.18
Y326$^{7.43}$		1.00		0.98	0.00		1.00		0.98	0.00
N328$^{7.45}$		0.06	0.94	0.84	0.61	0.11		0.89	0.62	0.07
N332$^{7.49}$		1.00		0.98	0.04		1.00		0.97	0.00
Y336$^{7.53}$		1.00		0.98	0.00		1.00		0.98	0.03
D340$^{8.47}$			1.00	0.98	0.00		1.00		0.97	0.04
I155$^{3.40}$	0.15	0.47	0.38	0.51	0.63		0.15	0.85	0.79	0.20
F289$^{6.44}$	0.02	0.02	0.96	0.90	0.09			1.00	0.93	0.03
W293$^{6.48}$	0.01	0.50	0.49	0.31	0.01		0.01	0.99	0.95	0.01

	with beta-arrestin-2									
	active					inactive				
	rotamer					rotamer				
residue	g+	g-	t	S^2	tr/ns†	g+	g-	t	S^2	tr/ns†
D114$^{2.50}$		1.00		0.99	0.00		0.99	0.01	0.97	0.06
R165$^{3.50}$		1.00		0.97	0.11		0.98	0.02	0.93	1.04
Y326$^{7.43}$		1.00		0.98	0.00		1.00		0.97	0.01
N328$^{7.45}$			1.00	0.97	0.13			0.99	0.95	0.03
N332$^{7.49}$		1.00		0.98	0.02		0.14	0.86	0.67	0.12
Y336$^{7.53}$		1.00		0.98	0.00		1.00		0.98	0.00
D340$^{8.47}$		0.34	0.66	0.48	0.26		0.33	0.67	0.31	0.24
I155$^{3.40}$	0.97	0.03		0.91	0.04	0.11	0.11	0.78	0.49	0.13
F289$^{6.44}$			1.00	0.98	0.00		0.96	0.04	0.98	0.01
W293$^{6.48}$	0.01	0.03	0.96	0.95	0.01	0.01	0.99		0.98	0.01

	with Nb39 nanobody									
	active					inactive				
	rotamer					rotamer				
residue	g+	g-	t	S^2	tr/ns†	g+	g-	t	S^2	tr/ns†
D114$^{2.50}$		1.00		0.98	0.00		1.00		0.98	0.01
R165$^{3.50}$		1.00		0.98	0.00		1.00		0.98	0.00
Y326$^{7.43}$		1.00		0.98	0.00		1.00		0.98	0.00
N328$^{7.45}$		0.01	0.99	0.94	0.34		0.18	0.82	0.57	0.22
N332$^{7.49}$		0.55	0.45	0.32	1.65		0.99	0.01	0.96	0.03
Y336$^{7.53}$		0.99	0.01	0.96	0.15		1.00		0.98	0.00
D340$^{8.47}$		0.10	0.90	0.75	0.07		1.00		0.97	0.01
I155$^{3.40}$		0.91	0.09	0.96	0.14		0.05	0.95	0.92	0.15
F289$^{6.44}$		0.01	0.99	0.96	0.00		0.03	0.97	0.94	0.17
W293$^{6.48}$		0.22	0.78	0.62	0.00	0.01	0.02	0.97	0.97	0.00

	with T4 lysozyme fusion									
	active					inactive				
	rotamer					rotamer				
residue	g+	g-	t	S^2	tr/ns†	g+	g-	t	S^2	tr/ns†
D114$^{2.50}$		1.00		0.98	0.00		0.94	0.06	0.88	0.10
R165$^{3.50}$		1.00		0.99	0.01		1.00		0.98	0.08
Y326$^{7.43}$		1.00		0.98	0.01		1.00		0.98	0.00
N328$^{7.45}$			1.00	0.96	0.09		1.00		0.87	0.21
N332$^{7.49}$		0.99	0.01	0.95	0.04		0.03	0.97	0.92	0.01
Y336$^{7.53}$		1.00		0.98	0.01		0.98	0.02	0.98	0.00
D340$^{8.47}$			1.00	0.98	0.01		1.00		0.96	0.00
I155$^{3.40}$		0.86	0.14	0.95	0.17	0.71	0.25	0.04	0.29	0.03
F289$^{6.44}$			1.00	0.97	0.01		0.88	0.12	0.97	0.02
W293$^{6.48}$		1.00		0.98	0.00		1.00		0.98	0.00

Data are shown only for populated rotameric states. Ballesteros-Weinstein numbering of residues is indicated in superscript.

† average number of transitions between rotameric states per nanosecond.

Table S3. Transmembrane residues of the active, EM2-bound, G protein-coupled MOP in contact with water molecules during simulation. Data include hydrogen bonds formed with the protein backbone. Residues of the proposed polar signaling channel are shown in red.

TM residues†	Motif	Water Occupancy‡ / %
N86$^{1.50}$	Allosteric BP	84.60
T103$^{2.39}$		96.85
N104$^{2.40}$		92.16
L110$^{2.46}$		67.40
D114$^{2.50}$	Allosteric BP	92.84
T118$^{2.54}$		6.85
T120$^{2.56}$		61.67
Q124$^{2.60}$	BP	94.07
Y128$^{2.64}$	BP	97.79
D147$^{3.32}$	BP	100.00
N150$^{3.35}$	BP	81.53
M151$^{3.36}$	BP	37.26
S154$^{3.39}$	Allosteric BP	93.85
L158$^{3.43}$		46.76
T160$^{3.45}$		30.25
D164$^{3.49}$	DRY	99.61
R165$^{3.50}$	DRY	99.94
R179^{ICL2}	ICL2	98.25
N188$^{4.46}$		74.32
N191$^{4.49}$		76.17
A240$^{5.46}$		78.36
Y252$^{5.58}$		93.41
C292$^{6.47}$	CWxP	82.03
W293$^{6.48}$	CWxP, BP	60.06
H297$^{6.52}$	BP	58.20
C321$^{7.38}$		71.68
I322$^{7.39}$	BP	15.14
Y326$^{7.43}$	BP	90.70
N328$^{7.45}$		95.62
S329$^{7.46}$		81.03
N332$^{7.49}$	NPxxY	95.81
L335$^{7.52}$	NPxxY	89.34
Y336$^{7.53}$	NPxxY	99.65
L339$^{7.56}$		92.92
D340$^{8.47}$		100.00
E341$^{8.48}$		99.99

† Ballesteros-Weinstein numbering is shown in superscript
‡ Occupancy represents the fraction of time the residue spent H-bonded to water during the simulation.
